# Effectiveness and safety of vitamin K antagonists and new anticoagulants in the prevention of thromboembolism in atrial fibrillation in older adults – a systematic review of reviews and the development of recommendations to reduce inappropriate prescribing

**DOI:** 10.1186/s12877-017-0573-6

**Published:** 2017-10-16

**Authors:** Christina Sommerauer, Lisa Schlender, Mark Krause, Sabine Weißbach, Anja Rieckert, Yolanda V Martinez, David Reeves, Anna Renom-Guiteras, Ilkka Kunnamo, Andreas Sönnichsen

**Affiliations:** 1 Institute of General Practice and Family Medicine, University of Witten/Herdecke, Alfred-Herrhausen-Straße 50, 58448 Witten, Germany; 2 NIHR School for Primary Care Research, Manchester Academic Health Science Centre, University of Manchester, Manchester, UK; 3 Department of Geriatrics, University Hospital Parc de Salut Mar, Barcelona, Spain; 4 Duodecim Medical Publications Ltd, Helsinki, Finland

**Keywords:** Atrial fibrillation, NOACs, VKA, Anticoagulation, Systematic review, Effectiveness, Safety, PRIMA-eDS, Treatment, Prevention, Thromboembolism, Stroke, Bleeding, Elderly

## Abstract

**Background:**

Oral anticoagulants are used for stroke prevention in patients with atrial fibrillation, the most common cardiac arrhythmia in older adults. The aim of our study was to identify the evidence on the risks and benefits of anticoagulant use among adults aged ≥65 years with atrial fibrillation and to develop recommendations to reduce inappropriate use with a primary focus on new oral anticoagulants.

**Methods:**

Systematic review (SR) with search in six databases (up to 12/2016). We included SRs/meta-analyses (MAs) with participants ≥65 years old with atrial fibrillation treated with oral anticoagulation. Two independent reviewers performed study selection, data extraction and quality appraisal. Recommendations were developed based on the evidence identified following a modified GRADE approach.

**Results:**

Thirty-eight SRs/MAs were included, drawing on evidence from 74 individual experimental studies. The mean age ranged from 68.2 to 73 years. Treatments investigated included vitamin K antagonists (VKA), new oral anticoagulants (NOACs), platelet aggregation inhibitors (PAI), placebo and no treatment. Comorbidities were reported in 23 SRs, but none reported on frailty status, cognitive status or polypharmacy. Sixteen SRs based on only 3–8 RCTs and thus conveying a significant overlap of studies evaluated the effectiveness of NOACs compared to warfarin. NOACs demonstrated at least equivalent ability to reduce stroke as VKA and a considerably lower risk (OR 0.37 to RR 0.50) of haemorrhagic stroke/intracranial bleeding. Seven SRs were identified comparing VKA to placebo. These revealed a substantial reduction in risk of stroke and mortality for VKA (RR 0.30–0.46)), outweighing an increased risk of bleeding (RR 1.04–3.63) associated with anticoagulation. Eight SRs evaluated the efficacy of VKA compared to PAI: overall, VKA were associated with a lower risk of stroke (OR 0.51–0.68)) and a comparable risk of major bleeding.

**Conclusions:**

Anticoagulation treatment using VKA in older people with atrial fibrillation appears beneficial in comparison to PAI and placebo. New oral anticoagulants appear to reduce haemorrhagic strokes and intracranial bleedings more effectively than VKAs and should be considered especially in patients with low TTR (time in therapeutic range)/labile INR (International Normalized Ratio). However, to determine if these results are applicable to all older people, further studies should provide information on frailty, significant impaired renal function, polypharmacy and cognitive status of the participants.

Funded by the 7th framework programme of the European Union.

## Background

Atrial fibrillation is the most common arrhythmia seen in clinical practice and its prevalence increases rapidly with age. Around 9% of people aged 80 years or older are affected by atrial fibrillation [1]. Atrial fibrillation is associated with a fivefold higher risk for stroke and with increased mortality [2, 3]. In the Framingham Heart Study atrial fibrillation was the only cardiovascular condition that showed greater risk of stroke with increasing age [3]. Anticoagulation with vitamin K antagonists (VKA) in atrial fibrillation has been the mainstay of therapy for decades to prevent stroke and systemic embolism [4, 5]. However, the need for regular monitoring of INR (International Normalized Ratio) and multiple drug and food interactions of VKA have led to the development of new oral anticoagulants (NOACs). NOACs are taken orally in a fixed regime once or twice daily without any need for laboratory measurements. A distinction can be made between direct thrombin inhibitors such as dabigatran, and direct factor Xa inhibitors such as rivaroxaban, apixaban and edoxaban. In the European Union in 2008 Dabigatran became the first NOAC approved for stroke prevention in atrial fibrillation. Since their approval, there has been some controversy over the use of NOACs, especially in older people, while prescription rates have continuously increased.

The European Society of Cardiology (ESC) Guidelines recommend NOACs for the majority of patients with AF as NOACs were effective in preventing stroke with better safety compared to VKA [6]. Elderly patients are at higher risk for stroke and therefore benefit from treatment with oral anticoagulation. However, concerns remain over patients with multiple comorbidities and polypharmacy as they are at higher risk for adverse drug events and drug interactions requiring dose-adjustments in this patient group [6]. NOACs are considered potentially inappropriate medications for older people by some authors [7]. In contrast, the National Institute for Health and Care Excellence (NICE) guidelines recommend the NOACs apixaban, rivaroxaban and dabigatran as equal to VKA [8].

The risk of major bleeding associated with use of anticoagulants remains a serious concern. Bleeding associated with warfarin is one of the main causes of adverse event related hospitalizations [9], with people aged 75 or older and with polypharmacy at particularly higher risk. The bleeding risk appears to be the main reason for VKA underuse in almost half of the patients with atrial fibrillation eligible for anticoagulation, and especially in older people [10, 11].

As a consequence of increasing life-expectancy and medical progress, multimorbidity and its corollary polypharmacy have been increasing in recent years and this is seen most distinctly in older adults [12, 13]. In addition to this, polypharmacy is very common among older people, with one third of adults aged 65 or older taking five drugs or more per day [14, 15]. Polypharmacy increases the risk of adverse events due to interactions and may not be appropriate for all patients.

The objectives of this review were to evaluate the risks and benefits of the use of oral anticoagulants in the treatment of atrial fibrillation in older adults and to use the evidence identified for the development of recommendations as to when and which anticoagulants should be preferred or discontinued in older people with atrial fibrillation. These recommendations will be implemented in an electronic decision support tool used to reduce polypharmacy in older adults in the project “Polypharmacy in chronic diseases: Reduction of Inappropriate Medication and Adverse drug events in elderly populations by electronic Decision Support” (PRIMA-eDS).

## Methods

We performed a systematic review (SR) of existing research literature on the risks and benefits of the use of oral anticoagulants in the treatment of atrial fibrillation in people aged 65 years or older.

### Search strategy

This SR is part of a series of SRs on the efficacy and safety of commonly prescribed drugs in older people. An efficient method based on the methods described in the Cochrane Handbook for Systematic Reviews of Interventions and the Preferred Reporting Items for Systematic Reviews and Meta-Analyses [16, 17] was used. This method has been published in detail [18]. Briefly, a four-stage approach was used:

- In search 1 and 2, SRs and MAs from a database search were retrieved.
- In search 3A, individual studies from not included SRs were retrieved.
- In search 3B, individual studies from a database search were retrieved.

Each subsequent search was only performed if the team of researchers decided that the so far accumulated evidence was not sufficient, or of sufficient quality, for evidence-based recommendations to be made.

A study protocol for this SR is available upon request from the authors.

Search 1 and 2 were performed on 5th October 2015 and an update was performed on 12th December 2016 by trained researchers at the University of Manchester and included the Cochrane Database of Systematic Reviews (CDSR), the Database of Abstracts of Reviews of Effects (DARE), MEDLINE, EMBASE, Health Technology Assessment Database (HTA) and International Pharmaceutical Abstracts (IPA), without any limitation on study publication dates. We considered that these searches yielded sufficient high quality evidence, making it unnecessary to conduct searches 3A or 3B for single studies. To be sure we did a comprehensive search on 2nd February 2017 with our existing search terms in the following databases: MEDLINE, EMBASE, Health Technology Assessment Database (INAHTA), Cochrane and DARE for 2015 till today. In these timeframe exactly 1615 hits emerged, which we all screened regarding our inclusion criteria adding a specific RCT criterion, with two independent reviewers checking. No new RCT-evidence has appeared. We checked 55 additional full texts for inclusion. Most of the texts were excluded because less than 80% of the patients were 65 years and above. For all databases we used the same search string based on the PICOS framework documented in Additional file 1. All duplicates were removed. All references from both searches were combined in one Endnote file. In addition to database searches, the references of included studies were checked to obtain a comprehensive list of studies. The citations were scrutinized and the full manuscripts were obtained for all citations potentially meeting the inclusion criteria. For pragmatic reasons the references of included lists were not systematically checked for duplicates.

### Study selection

Two reviewers performed study selection of titles/abstracts and full-texts independently by using the following a priori defined criteria. Disagreements were resolved through discussion and by arbitration by a third reviewer if necessary.

### Inclusion criteria for systematic reviews and meta-analyses

- SRs evaluating benefits and/or risks of VKA and/or new anticoagulants in the treatment/prevention of thromboembolism in atrial fibrillation
- Mean age ≥ 65 years or more than 80% of the studies reporting a mean age ≥ 65 years OR mean age < 65 but subgroup analysis reporting on participants ≥65 years
- Clinically relevant endpoints of effectiveness: mortality, stroke, systemic embolism (SE), ischemic stroke (IS), haemorrhagic stroke (HS), myocardial infarction (MI) OR clinically relevant safety endpoints: major-, intracranial- and gastrointestinal bleedings.

### Exclusion criteria

- Pooled analyses not based on a systematic literature search
- Narrative reviews, editorials, opinion papers, letters, proceeding and conference papers
- More than 50% of included studies phase II studies or drugs that were not approved in the European Union at time of performance of our review (December 2016)

Details of excluded studies and reasons for exclusion are provided by the authors on request.

### Types of interventions

We included studies reporting on the efficacy and/or safety of any oral anticoagulants for the management of atrial fibrillation including vitamin K antagonist and novel oral anticoagulants. We included studies comparing oral anticoagulation with placebo, no treatment, and other drugs including platelet aggregation inhibitors (PAI).

### Types of outcomes

We included clinically relevant endpoints of effectiveness and safety such as mortality, any stroke, systemic embolism (SE), ischemic stroke (IS), haemorrhagic stroke (HS), myocardial infarction (MI), major bleeding, intracranial bleeding (including intraparenchymal bleedings) and gastrointestinal bleedings.

### Data extraction and quality appraisal

For each included publication, both reviewers used a standardised and piloted data extraction sheet to independently extract all data, with disagreements resolved by discussion. Study quality was also assessed independently by two reviewers using a reliable and validated measurement tool to assess the methodological quality of systematic reviews (AMSTAR) [19, 20]. Because there are no clear recommendations on how to report the results of the AMSTAR quality appraisal tool, we decided to report the data in a descriptive way.

Additionally, we collected information on the quality of the individual studies of the SRs included and used for the recommendations. If this information was not available, we performed quality appraisal of the individual studies using the Cochrane tool for quality appraisal of clinical trials if not done by the included SRs [16].

### Identification of additional references of interest

During the process of study selection we also looked for papers of interest that were not part of our systematic review, but that might still inform the development of recommendations, following our study protocol [18].

### Development of recommendations

Based on all the identified evidence the reviewers developed recommendations for the use of VKAs and NOACs in older people. Each recommendation was given a rating for strength (weak or strong) and for quality of evidence (low, moderate or high), following the Grading of Recommendations Assessment, Development and Evaluation (GRADE) methodology [21–23]. For reason of simplification we used only three categories for the quality of evidence, following the American College of Physicians’ Guideline Grading System [24]. As these recommendations are used in the PRIMA-eDS-tool to reduce polypharmacy and inappropriate prescribing, we used the evidence identified for the development of recommendations as to when and which anticoagulants should be preferred or discontinued in older people. The suggestions for recommendations were discussed and approved by an editorial board for the development of evidence based medicine (EBM) guidelines and recommendations of Duodecim Medical Publication Ltd. from Finland. The Editorial Team of EBM Guidelines consists of 10 members including eight physicians (six general practitioners, one neurologist and one specialist in internal medicine and oncology). Additionally, there are permanent experts including one pulmonologist, one urologist and one otorhinolaryngologist. The decision support rules are finalized by the Editorial Team of the EBMeDS decision support service including 10 members, of which four are also members of the EBM Guidelines Editorial Team or Editorial Board. The Editorial Team of EBMeds include four general practitioners, one specialist in internal medicine and infectious diseases and one nurse. Additionally, four members of the EBM Guidelines Editorial Team serve as advisors and referees for EBMeDS contents. The members of the teams do not have conflicts of interests [25].

## Results

We performed searches 1 and 2. The identified evidence was judged to be sufficient and of sufficient quality to develop. We expected no relevant current studies for the comparison between VKA and placebo or for the comparison between VKA and PAI. The comparison of VKA and NOACs is a very current topic but we expected all relevant RCTs to be included in our included systematic reviews. In addition, we screened clinicaltrials.gov. We identified a phase 2 study of betrixaban but this oral anticoagulants is not be expected to be approved within the next year for the indication of atrial fibrillation.

**Fig. 1:**
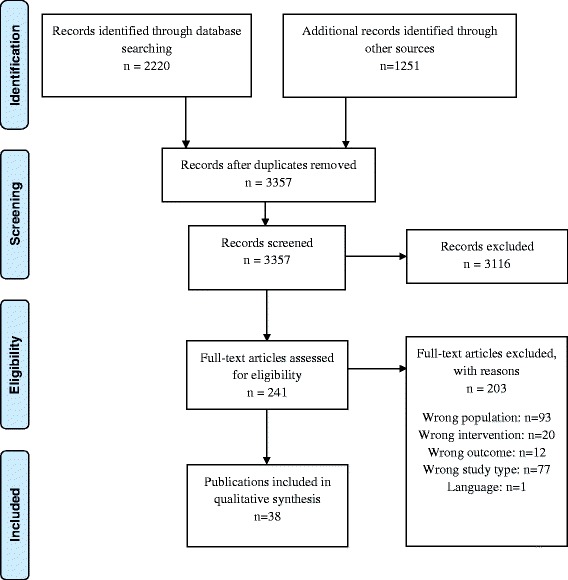
Preferred Reporting Items for Systematic Reviews and Meta-Analyses (PRISMA) flow diagram

We identified 605 references in searches 1 and 2 and 1615 references in the comprehensive search. Another 1251 references were identified by screening the reference lists of included articles and by hand-searching. After removal of duplicates, we screened a total of 3357 references. Of these, we obtained and assessed 241 full texts against our inclusion and exclusion criteria, and subsequently excluded 203 of these. This left a total of 38 systematic reviews providing evidence relevant to our purpose. The process of study selection is displayed in Fig. 1 (PRISMA flow chart).

The main characteristics of all included SRs are presented in Table 1. The included SRs were published between 1999 and 2015. The number of included studies ranged from 3 to 49 and the number of included participants from 1940 to 897,748. The lowest mean age was 68.2 years and the highest 73 years. Five SRs provided additional data on subgroups for people aged ≥75 years [26–30]. Four SRs included studies of people with AF and with venous thromboembolism and provided subgroup data for people with AF [26, 31–33]. Follow up time was at least 1 year in all but five SRs. Intervention- and control-treatments included VKA (warfarin, acenocoumarol), NOACs (apixaban, dabigatran, edoxaban, rivaroxaban, ximelagatran), PAI (acetylsalicyl acid (ASA), clopidogrel, triflusal, indobufen), placebo and no treatment.

**Table 1:** Characteristics of included systematic reviews

Source	Type of study	Includes AF-studies N	AF-patients N	Mean age in years	Intervention	Comparator	mean Follow-Up in years	Relevant outcomes							
								AS	IS	HS	M	MB	IB	GB	MI
Adam et al. 2012 [31]	MA	3	50,578	>70	NOACs (dabigatran, apixaban, rivaroxaban)	Warfarin	≈ 2		X	X	X				
Agarwal et al. 2012 [27]	MA	8	32,053	70–81.5 subgroup ≥75	Warfarin	Other thrombophylaxis (Aspirin, Aspirin + clopidogrel, Ximelagatran, Dabigatran, Rivaroxaban, Apixaban, Indraparinux)	1.74^a^	X							
Aguilar et al. 2005 [34]	CR + MA	5	2313	69	Warfarin	Placebo/control	1.5	X	X		X	X	X		X
Aguilar et al. 2007 [44]	MA	8	9598	71	Warfarin, acenocumarol	PAI (ASA, ASA + clopidogrel, triflusal)	1.9	X	X		X	X	X		X
Andersen et al. 2008 [37]	MA	15	16,058	63.3–81.5	warfarin	Placebo, antiplatelets, low dose warfarin, low-dose warfarin + aspirin	1.7					X			
Assiri et al. 2013 [42]	MTM	21	80,906	71	Pair-wise comparison of aspirin, aspirin + clopidogrel, warfarin, apixaban, dabigatran, edoxaban, rivaroxaban and placebo		1.85^a^	X			X	X	X		
Baker et al. 2012 [53]	MA	4	44,733	69–73	NOACs (dabigatran, apixaban, rivaroxaban)	Warfarin indirect comparison of NOACs	1.8–2.0	X	X	X	X	X		X	
Briceno 2015 [29]	MA	7	73,978	70–74	NOACs (dabigatran, rivaroxaban, apixaban, edoxaban) DEVICE (Watchman left atrial occlusion device)	Warfarin	1–2.8	X			X	X			
Cameron et al. 2014 [46]	MTM	16	82,314	62–83	VKA	NOACs ASA	12 weeks −3.5 years	X				X			
Capodanno et al. 2013 [47]	MA	3	50,578	71.3	NOACs (dabigatran, apixaban, rivaroxaban)	Warfarin	1.9	X	X	X	X	X	X	X	X
Chatterje et al. 2013 [63]	MA, MTM	6	57,491	69.3–73	NOACs (dabigatran, apixaban, rivaroxaban)	Warfarin, ASA	0.23–2.0						X		
Coleman et al. 2012 [35]	MA	16	42,983	69.5	Pair-wise comparisons of warfarin (adjusted + low dose), PAI, ximelagatran, dabigatran, placebo/control		2							X	
Cooper et al. 2006 [41]	MTM	19	17,833	68.3	Pair-wise comparison of warfarin (adjusted + low dose), ASA, indobufen, ximelagtran, placebo/control		1.8		X			X			
Dogliotti et al. 2013 [48]	MA	5	51,895	71.4	NOACs (dabigatran, apixaban, rivaroxaban, ximelagatran)	Warfarin	1.75	X	X	X	X	X			
Dogliotti et al. 2014 [40]	MTM	20	79,808	68.2	Pair-wise comparison of ASA, ASA plus clopidogrel, VKA, dabigatran, rivaroxaban, apixaban or placebo/control		>1	X	X		X	X			
Harenberg et al. 2012 [70]	MTM	3	50,578	70–73	NOACs (dabigatran, apixaban, rivaroxaban)	Warfarin	1.88		X		X	X	X		X
Hart et al. 1999 [4]	MA	6	9874	70	Adjusted-dose warfarin, PAI	Other Antithrombotic regimens placebo ASA	Mean FU 1.7 y1.7ears, ranged from 1.2 to 2.3	X	X		X	X	X		
Hart et al. 2007 [39]	MA	29	28,044	71	Adjusted-dose warfarin,	PAI Placebo/No Treatment	1.5	X	X		X	X	X		
Holster 2013 [33]	MA	8	151,578	65–73	NOACs (apixaban, edoxaban, rivaroxaban, dabigatran)	Warfarin	12 weeks – 2 years					X		X	
Jia 2014 [54]	MA	5	72,961	70–73	NOACs (apixaban, edoxaban, rivaroxaban, dabigatran)	Warfarin	1.6–2.8	X	X	X	X	X	X	X	X
Lega 2014 [30]	MA	3	50,578	70–73	NOACs (apixaban,rivaroxaban, dabigatran)	Warfarin	1.8–2	X				X			
Liew et al. 2014 [49]	MA	4	71,683	no mean	NOACs (dabigatran, apixaban, rivaroxaban, edoxaban)	Warfarin	1.8–2.8				X		X		
Lin 2015 [43]	MTM	49	897,748	71	Warfarin	ASA, dabigatran, rivaroxaban, apixaban, edoxaban, no treatment	12 weeks −3.6 years	X	X		X	X	X	X	
Lip et al. 2006 [36]	MA	13	14,423	68.4	Adjusted-dose warfarin	ASA fixed low-dose warfarin, placebo, ximelagatran	2.13		X		X	X			
Miller et al. 2012 [50]	MA	3	44,563	71.5	NOACs (dabigatran, apixaban, rivaroxaban)	Warfarin	1.91	X	X	X	X	X	X	X	X
Providência et al. 2014 [51]	MA	7	80,290	71.3	NOACs (dabigatran, apixaban, rivaroxaban, ximelagatran, edoxaban)	Warfarin	1.96	X	X		X	X	X	X	X
Rong 2015 [66]	MA	4	71,683	70–73	NOACs (apixaban, edoxaban, rivaroxaban, dabigatran)	Warfarin	1.8–2.8					X	X	X	
Roskell et al. 2010 [67]	MTM	26	not stated	71.2	dabigatran	Indirect comparison: Placebo, ASA, ASA + clopidgrel, adj. Dose VKA	1.87	X	X		X	X	X		X
Ruff et al. 2014 [87]	MA	4	71,683	71.4	NOACs (dabigatran, apixaban, rivaroxaban, edoxaban)	Warfarin	2	X	X	X	X	X	X	X	X
Sardar et al. 2013 [64]	MA	3	50,578	70.6	NOACs (dabigatran, apixaban, rivaroxaban)	Warfarin	1.88	X		X	X	X	X	X	-
Sardar et al. 2014 [32]	MA	4	11,562	subgroup for ≥75	NOACs (dabigatran, apixaban, rivaroxaban)	Warfarin, ASA	1.1–2.0	X				X			
Schneeweiss et al. 2012 [69]	MTM	3	44,535	70–73	NOACs (dabigatran, apixaban, rivaroxaban)	Warfarin	1.8–2.0	X				X			
Segal et al. 2000 [38]	MA	11	8690	66–80	VKA	ASA ASA + low dose warfarin placebo	0.3–2.6	X			X	X			
Senoo 2015 [65]	MA	3	1940	70–73	NOAC (rivaroxaban, apixaban, dabigatran)	Warfarin	1.3–2.0	X				X	X	X	
Sharma et al. 2015 [26] (Subgroup >75 with AF)	MA	5	27,622	>75	Single NOACs (dabigatran, apixaban, rivaroxaban, edoxaban)	Warfarin	1.75	X			X				
Taylor et al. 2001 [45]		5	3298	73	Warfarin	PAI	2.35	X			X	X			X
Testa et al. 2012 [52]	MA	3	50,578	71.5	NOACs (dabigatran, apixaban, rivaroxaban)	Warfarin	>1	X	X	X	X	X			X
Verdecchia et al. 2015 [68]	MTM	4	71,683	71.5	Indirect comparison (dabigatran, apixaban, rivaroxaban, edoxaban)	Dose-adjusted warfarin	2.15	X	X		X	X	X	X	X

*AS* All stroke, *CR* Cochrane Review, *GB* Gastrointestinal Bleeding, *HS* Haemorrhagic Stroke, *IB* Intracranial Bleeding, *IS* Ischemic stroke, *M* Mortality, *MA* Meta-analysis, *MB* Major Bleeding, *MI* Myocardial infarction, *MA* Meta-analysis, *NMA* network meta-analysis

^a^ Calculated by the review team

No SR reported on the number of participants with polypharmacy or on the functional or cognitive status of the participants. Comorbidities were reported in 23 SRs. Hypertension, diabetes, prior myocardial infarction and prior stroke were the comorbidities most frequently reported. All SRs that provided data about prior stroke included studies of both primary and secondary stroke prevention. The CHADS_2_-score was reported by 21 SRs. Time in therapeutic range (TTR) was reported by 27 SRs and ranged from 42 to 84% in the single studies. For the comparison between NOACs and VKA, the TTR ranged from 44 to 68%. The characteristics of the participants of all the included SRs are summarised in Additional file 2: Table S1. The dates and data base searches of the individual systematic reviews are shown in Additional file 3: Table S2.

### VKA vs. Placebo

We identified seven SRs that examined the effectiveness of warfarin compared to placebo [4, 34–39]. These seven SRs in combination included a total of six different original studies. The SRs varied considerably in respect to the effect models (fixed-effect or random-effect) and effect measures (odds-ratio, relative risk, or relative risk reduction) used. In addition, we included three SRs that used mixed treatment comparisons including a comparison of warfarin vs. placebo [40–42] The NMA by Lin et al. [43] compared warfarin to no treatment and included also non-randomised trials. For a better comparability, results of the meta-analysis for RCTs only are described. The results are shown in Table S3 (Additional file 4) of the Additional files.

#### Effectiveness outcomes

##### Stroke/SE

Three out of seven SRs reported on stroke/systemic embolism as an outcome and one only on systemic embolism [37]. All reported an advantage for VKA compared to placebo. Aguilar et al. [34] and Segal et al. [38] included the same subset of studies and found a large reduction in stroke events associated with warfarin compared to placebo, with an OR of 0.39 (95% CI 0.26–0.59) and an OR of 0.30 (95% CI 0.19–0.48). Hart et al. [4] included additionally the EAFT study and reported a relative risk reduction (RRR) for all stroke events of 62% (48–72%) for warfarin and a RRR of 64% (95% CI 49%–74%). Hart et al. [39] added 13 RCTs in an update, but no additional comparisons of warfarin vs. placebo were included. Andersen et al. reported on SE only and the direction of effect favoured warfarin [37]. The NMAs supported these results and reported fewer stroke events with warfarin than with placebo [40, 42, 43].

##### Ischemic stroke

Four SRs investigated ischemic stroke and three included the same subset of five studies. All produced similar effect estimates in favour of warfarin. Aguilar et al. [34] calculated an OR of 0.34 (95% CI 0.23–0.52), similarly to Lip et al. [36] who included one study more in their SR (RR 0.33, 95% CI 0.24–0.45). Hart et al. [4] reported an RRR of 65% (95% CI 52%–74%) associated with warfarin, and Hart et al. [39] an RRR of 67% (95% CI 54%–77%).

Three NMAs reported on ischemic strokes and found likewise a reduced risk of ischemic strokes for VKA vs. placebo/no treatment [40, 41, 43] lin.

##### Haemorrhagic stroke

No SR reported on this outcome.

##### Mortality

The inlcuded SRs found a substantial effect in favour of warfarin, including an OR of 0.69 (95% CI 0.50–0.94) [34] and an RR of 0.69 (95% CI 0.53–0.89) [36]). In Hart et al. [4], warfarin was associated with a significant RRR of 26% (95% CI 4%–43%) for mortality, a result repeated in the review update in 2007 based on the same set of studies [39]., Segal et al. [38] found a point estimate of effect that was similar to the other SRs (OR 0.62, 95% CI 0.38–1.02). Two of the NMAs also found VKA (mostly warfarin) to be associated with reduced risk of mortality (RR 0.60, 95% CI 0.43–0.77 [40] and RR 0.67, 95% CI 0.50–0.89) [42]).

#### Safety outcomes

##### Major bleeding

Six SRs reported on major bleeding but differed in the definition of this outcome. Aguilar et al. [34], Hart et al. [4] and Hart et al. [39] considered extracranial major bleeding only, while Lip et al. [36], Andersen et al. [37] and Segal et al. [38] examined all major bleeding. Aguilar et al. [34] found no difference between warfarin and placebo while Segal et al. found a higher risk for warfarin [38]. In the reviews by Andersen et al. [37] and Lip et al. [36] warfarin was associated with a considerably increased risk of bleeding (OR 3.01, 95% CI 1.31–6.92; and RR 0.45, 95% CI 0.25–0.82, respectively) [37]. Hart et al. [4] likewise found an association between VKA and a higher risk of extra-cranial bleeding (RR 2.4, 95% CI 1.2–4.6). However, in the update using the same set of studies a RRR of −66% (95% CI -235 to 18%) was reported [39] (de facto a risk increase of 66%).

The NMA by Dogliotti et al. [40] used the trial-specific definition of the included studies for major bleeding and Cooper et al. [41] reported on major and fatal bleeding episodes. Dogliotti et al. [40] found a higher risk of bleeding for VKA vs. placebo (OR 3.63, 95% CI 1.84–9.06) as did Assiri et al. (RR 3.12 (1.05–9.96)) [42], whereas Cooper et al. [41] reported only a non-significant difference and Lin et al. [43] found only a slightly increased risk (RR1.14 (0.46–2.78)).

##### Intracranial bleeding

Only two SRs examined intracranial bleeding. Aguilar et al. [34] found a trend favouring placebo (OR 2.38, 95% CI 0.54–10.5). Hart et al. [4] found a low overall incidence of intracranial haemorrhage, with six cases in warfarin patients compared to three in those on placebo (not significantly different). The NMA by Lin et al. [43] showed a trend in favour of placebo (RR 1.25 (0.98–1.59)).

##### Gastrointestinal bleeding

Only one SR reported on gastrointestinal bleeding, the risk of which was comparatively greater for warfarin than for placebo (OR 3.21, 95% CI 1.32–7.82) [35]. The NMA by Lin et al. [43] found an increased risk for warfarin which was not significant with wide confidence intervals (RR 6.66 (0.28–100)).

##### Myocardial infarction

Only one SR reported data on myocardial infarction. Aguilar et al. [34] found a trend in favour of warfarin (OR 0.87, 95% CI 0.32–2.42), likewise the NMA by Lin et al. [43].

### VKAs vs. PAIs

We identified eight SRs that included a comparison between VKA and PAI [4, 35–39, 44, 45]. Between them, these eight SRs included 13 unique original studies. Most of the individual studies used ASA as the antiplatelet drug. Additionally, five SRs performed a NMA including a comparison of VKA vs. PAI [40–43, 46]. For a better comparability, results of the meta-analysis for RCTs only are described for Lin et al. [43]. The results of these 13 SRs are displayed in Additional file 4: Table S4.

There was one additional SR comparing warfarin to any other antithrombotic treatment including PAI but also NOACs [27]. We did not consider this SR in our analysis because it was not possible to differentiate between the effects of PAI and NOACs.

#### Effectiveness outcomes

##### Stroke/SE

The included SRs reporting on the outcome stroke found an advantage for warfarin compared to PAI [4, 38, 39, 44, 45]. Hart et al. [4] reported a reduction of stroke by 36% (95% CI 14%–52%) for warfarin. In the update of the review in 2007, four additional studies were included for the comparison of warfarin vs. PAI but with similar results for the outcome stroke/SE (RRR 37% (95% CI 23 to 48%)) [39]. Aguilar et al. [44] and Segal et al. [38] reported a reduction of stroke for warfarin (OR 0.68, 95% CI 0.54–0.85, and 0.64, 95% CI 0.43–0.96, respectively). Taylor et al. [45] found a trend for warfarin compared to PAI for fatal stroke (OR 0.74, 95% CI 0.39–1.40) and a distinct advantage for non-fatal stroke (OR 0.68, 95% CI 0.46–0.99).

Andersen et al. [37] reported only SE and found a advantage for warfarin compared to PAI (OR 0.50, 95% CI 0.33–0.75).

##### Ischemic stroke

Four SRs reported on ischemic stroke and found effects for warfarin compared to PAI (OR 0.53, 95% CI 0.41–0.68 [44]; RR 0.59, 95% CI 0.40–0.86 [36]; RRR 46%, 95% CI 27–60% [4]; and 52%, 95% CI 41 to 62% [39]).

The included NMAs found likewise a higher risk for ischemic stroke associated with ASA compared to warfarin (RR 1.85, 95% CI 1.25–2.58 [41]) and likewise a lower risk for warfarin compared to ASA [40, 43]. Dogliotti et al. found in their NMA a lower risk of all strokes for warfarin compared with PAI (OR 0.51, 95% CI 0.41–0.65) [40]. Assiri et al. [42] compared warfarin to ASA and to ASA plus clopidogrel and found for both comparisons a reduction of stroke associated with warfarin (compared to ASA RR 0.43 (95% CI 0.33–0.57), compared to ASA + clopidogrel: 0.60 (95% CI 0.42–0.85)). Cameron et al. [46] found a similar result for warfarin compared to ASA + clopidogrel (OR 0.52, 95% CI 0.38–0.70) and for warfarin compared to low dose ASA (<100 mg) (OR 0.53, 95% CI 0.36–0.79).

##### Mortality

Six SRs provided data on risk of mortality [4, 36, 38, 39, 44, 45]. All six found no significant difference between VKAs and PAIs (OR 0.99, 95% CI 0.83–1.18 [44]; OR 0.96, 95% CI 0.58–1.58 [38]; OR 0.83, 95% CI 0.46–1.50 [45]; RR 0.87, 95% CI 0.67–1.13 [36]; and RRR 8%, 95% CI –21 to 30%) [4]; and RRR 9%, 95% CI –19 to 30 [39]).

The NMA by Dogliotti et al. [40] favoured VKAs (OR 0.77, 95% CI 0.58–0.98). However, Assiri et al. [42] and Lin et al. [43] found only a trend in favour of warfarin compared to ASA (RR 0.85, 95% CI 0.70–1.02 and RR 0.94 (0.72–1.23)) or to ASA plus clopidogrel (RR 0.90, 95% CI 0.70–1.18).

#### Safety outcomes

##### Major bleeding

Seven SRs reported on major bleeding [4, 36–39, 44, 45]. One SR reported significantly decreased major bleeding with ASA compared to warfarin (RR 0.58, 95% CI 0.35–0.97) [36]. Three SRs reported no significant difference between VKA and PAI regarding risk of major bleeding [37, 38, 45]. Three reviews focused on extracranial haemorrhage [4, 39, 44]. Aguilar et al. [44] showed no significant difference comparing VKA to PAI (OR 0.97, 95% CI 0.74–1.28), while Hart et al. [4] found an increased risk of extracranial haemorrhage for warfarin compared to ASA (RR 2.0 (1.2–3.4)). However, in the update of 2007 including four additional studies, the difference was not significant (RRR –70%, 95% CI –234 to 14%) [39] (= a risk increase of 70%).

The NMA of Dogliotti et al. [40] reported an increased risk of major bleeding for VKA compared to ASA (OR 1.71, 95% CI 1.05–3.23), while Cooper et al. [41], Lin et al. [43] and Assiri et al. [42] found no significant differences. Three NMA compared warfarin with ASA plus clopidogrel and found no significant differences regarding major bleeding [42, 43, 46]). Cameron et al. [46] showed no difference in major bleeding for warfarin compared to low dose ASA (OR 0.95, 95% CI 0.53–1.67) and a non-significant reduction compared to ASA 100–300 mg (OR 0.56, 95% CI 0.18–1.61).

##### Intracranial and gastrointestinal bleeding

We identified three SRs reporting on intracranial bleeding. Aguilar et al. [44] reported a doubling of the risk for PAI compared to VKA (OR 1.98, 95% CI 1.20–3.28) and likewise Hart et al. [4] found twice as many intracranial haemorrhages with warfarin compared to ASA (RR 2.1, 95% CI 1.0–4.6), with a similar result in the update of 2007 (RRR –128%, 95% CI –399% to −4%) (= a risk increase of 128%) [39]. Coleman et al. also reported an increased rate of gastrointestinal bleedings with VKAs compared to PAIs (OR 1.92, 95% CI 1.08–3.41) [35].

The NMA of Assiri et al. [42] showed no difference for warfarin vs. ASA plus clopidogrel (rate ratio 1.03 (0.15–7.59)) and a non-significant increase of intracranial bleeding with warfarin compared to ASA (rate ratio 1.95 (0.45–9.29)), both with wide confidence intervals.

##### Myocardial infarction

Two SRs reported on myocardial infarction and found a trend in favour of warfarin. Aguilar et al. [44] reported an OR of 0.69 (95% CI 0.47–1.01) and Taylor et al. [45] an OR of 0.83 (95% CI 0.46–1.50), neither showing a significant advantage for either VKAs or PAI.

### NOACs vs. VKAs

We identified sixteen SRs comparing NOACs to warfarin [28, 29, 31–33, 47–54]. All reviews performed a quantitative synthesis. There was high overlap between the SRs in the studies included, with a total of eight unique original studies represented. All sixteen SRs included publications related to three registered trials of dabigatran, apixaban and rivaroxaban (RE-LY, ARISTOTLE and ROCKET AF, respectively) [55–57], and six included no further studies aside from these three. The other SRs additionally included the SPORTIF III and SPORTIF V (ximelagatran), ENGAGE AF-TIMI 48 (edoxaban), J-ROCKET AF (rivaroxaban) and/or PETRO (dabigatran) [58–62] trials. The doses of the NOACs reported correspond to the doses used in the single trials: dabigatran 2 × 150mg/day and dabigatran 2 × 110mg/day, apixaban 2 × 5mg/d, rivaroxaban 20 mg/day, edoxaban 2 × 60mg/day and edoxaban 2 × 30mg/day. All SRs were very similar in their inclusion and exclusion criteria and the research questions they addressed. However, there was some heterogeneity in the outcomes included, especially for the outcomes major bleeding and gastrointestinal bleeding (see Additional file 5: Table S5). All authors used a random effect model for their meta-analyses with the exception of Testa et al. [52]. There were three other SRs comparing either warfarin to any other antithrombotic treatment including NOACs [27] or comparing NOACs to any other antithrombotic treatment [32, 63]. We did not consider any of these reviews in this analysis because the comparator arm included a mix of either NOACs and PAI or VKA and PAI making it impossible to differentiate between the two. The event rates for each systematic review is summarised in Additional file 6: Table S6.

#### Effectiveness outcomes

Most of the SRs reported on outcomes of stroke/systemic embolism (SE), ischemic stroke, haemorrhagic stroke, mortality and myocardial infarction. For each outcome we summarised the pooled estimates of treatment effects from the meta-analyses in the different studies and conducted a qualitative synthesis of these results. Many of the SRs included a number of the same individual original studies, making it inappropriate to attempt to combine across them to obtain a global estimate of effect.

##### Stroke/SE

**Fig. 2:**
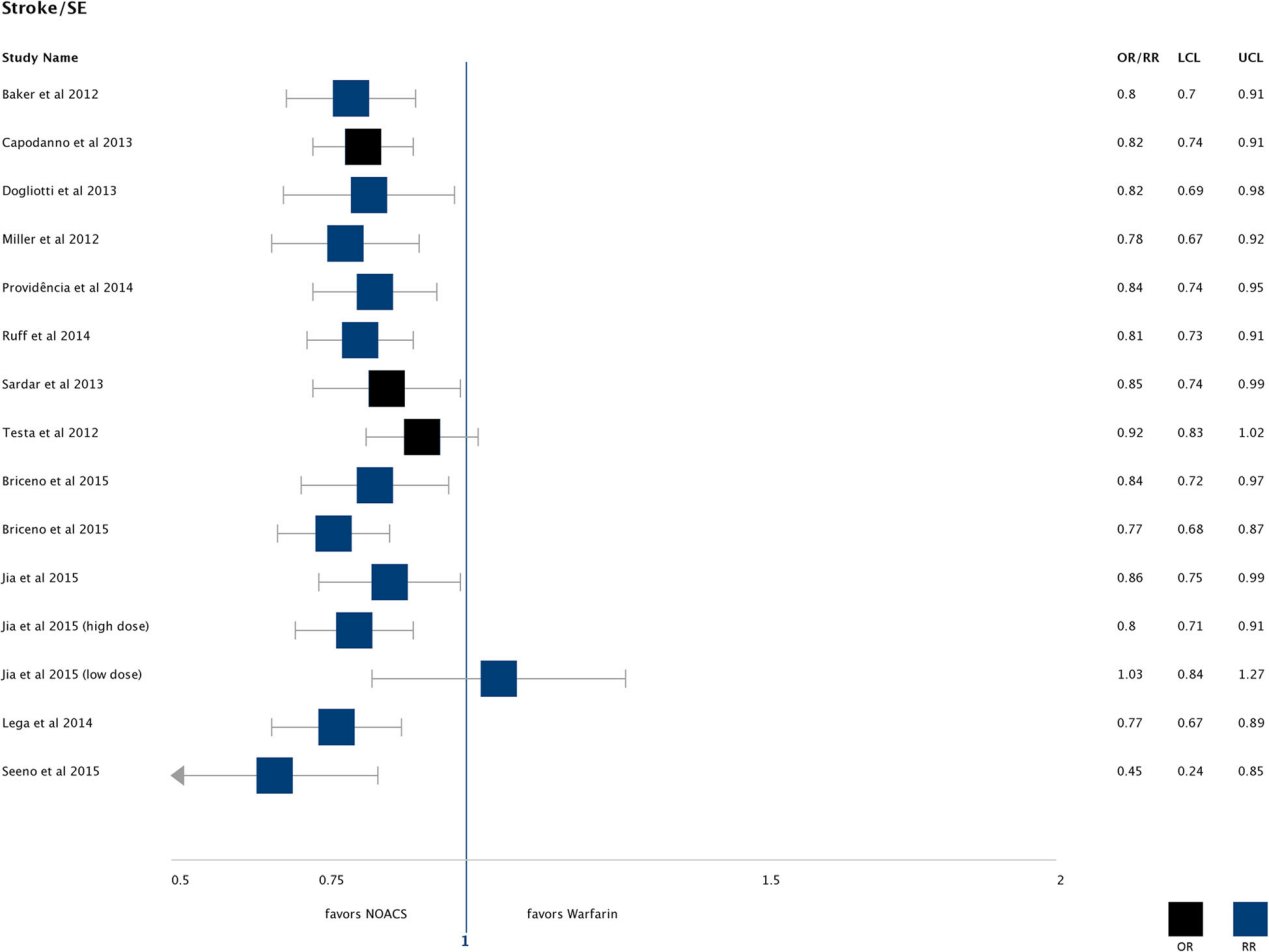
Stroke/SE: Found effects favouring NOACs compared to warfarin

Twelve SRs reported stroke/SE as an outcome [28–30, 47, 48, 50–54, 64, 65]. All found effects favouring NOACs compared to warfarin except the subgroup for low dose NOACs in the SR of Jia et al. [54] (see Fig. 2 and Table 2). Senoo et al. [65] showed a substantial advantage for NOACs (RR 0.45 (95% CI 0.24–0.85)) but included only Japanese patients and the smallest overall number of patients.

**Table 2:** Summary of study findings NOACs vs. VKA

	N studies	Effect- model	Effect measure	Stroke/SE	Ischemic stroke	Haemorrhagic stroke	Mortality	Major bleeding	Intracranial bleeding	Gastrointestinal bleeding	Myocardial infarction
Adam et al. 2012 [31]	3	REM	RR	Not stated	0.89 (0.78–1.02)	0.48 (0.36–0.62)	0.8 (0.82–0.96)	Not stated	Not stated	Not stated	Not stated
Baker et al.2012 [53]	4	REM	RR	0.797 (99% CI 0.695–0.914)	0.880 (99% CI 0.742–1.044)	0.445 (99% CI 0.269–0.768)	RR: 0.874 (99% CI 0.803–0.974)	0.878 (99% CI 0.664–1.160)	Not stated	1.254 (99% CI 0.827–1.901)	Not stated
Briceno et al. [29]	5	REM	OR	0.84 (0.72–0.97) subgroup >75 years: 0.77 (0.68–0.87)	Not stated	Not stated	0.89 (0.84–0.94)	0.794 (0.647–0.973)	Not stated	Not stated	Not stated
Capodanno et al. 2013 [47]	3	REM	OR	0.82 (0.74–0.91)	0.93 (0.82–1.05)	0.44 (0.30–0.66)	0.88 (0.82–0.95)	0.85 (0.69–1.05)	0.46 (0.38–0.55)	1.68 (1.03–2.72)	0.99 (0.71–1.38)
Dogliotti et al. 2013 [48]	5	REM	RR	0.82 (0.69–0.98)	0.87 (0.72–1.06)	0.49 (0.35–0.70)	0.91 (0.85–0.96)	0.84 (0.70–1.00)	Not stated	Not stated	Not stated
Holster et al. 2013 [33]	8	REM	OR	Not stated	Not stated	Not stated	Not stated	0.93 (0.75–1.16)	Not stated	1.21 (0.91–1.61)	Not stated
Jia et al. [54]	5	REM	RR	0.86 (0.75–0.99) high dose 0.80 (0.71–0.91) low dose 1.03 (0.84–1.27)	high dose 0.93 (0.84–1.03) low dose 1.13 (1.14–1.49)	high dose 0.50 (0.41–0.62) low dose 0.33 (0.23–0.46)	high dose 0.90 (0.85–0.95) low dose 0.89 (0.83–0.96)	0.78 (0.64–0.94) high dose 0.86 (0.74–0.99) low dose 0.63 (0.38–1.04)	high dose 0.48 (0.41–0.56) low dose 0.31 (0.24–0.41)	high dose 1.24 (1.10–1.39) low dose 0.52 (0.19–1.00)	high dose 0.97 (0.85–1.11) low dose 1.25 (1.04–1.50)
Lega et al. [30]	3	REM	RR	>75 years: 0.77 (0.67–0.89) <75 years: 0.83 (0.71–0.96)	Not stated	Not stated	Not stated	>75 years: 0.90 (0.82–1.00) <75 years: 0.73 (0.65–0.81)	Not stated	Not stated	Not stated
Liew et al. 2014 [49]	4	REM	RR	Not stated	Not stated	Not stated	0.89 (0.85–0.94)	Not stated	0.42 (0.34–0.53)	Not stated	Not stated
Miller et al. 2012 [50]	3	REM	RR	0.78 (0.67–0.92)	0.87 (0.77–0.99)	0.45 (0.31–0.68)	0.88 (0.82–0.95)	0.88 (0.71–1.09)	0.49 (0.36–0.66)	1.25 (0.91–1.72)	0.96 (0.73–1.26)
Providência et al. 2014 [51]	7	REM	RR	0.84 (0.74–0.95)	0.97 (0.83–1.14)	Not stated	0.90 (0.86–0.95)	0.79 (0.67–0.93)	0.49 (0.37–0.63)	1.07 (0.86–1.34)	1.01 (0.83–1.24)
Rong et al. 2015 [66]	4	REM	RR	Not stated	Not stated	Not stated	Not stated	0.77 (0.63–0.95) high dose: 0.86 (0.73–1.01) low dose: 0.63 (0.38–1.04)	0.42 (0.34–0.52) high dose: 0.48 (0.39–0.59) low dose: 0.31 (0.24–0.41)	1.10 (0.86–1.41) high dose: 1.25 (0.99–1.57) low dose: 0.87 (0.54–1.40)	Not stated
Ruff et al. 2014 [28]	4	REM	RR	0.81 (0.73–0.91)	0.92 (0.83–1.02)	0.49 (0.38–0.64)	0.90 (0.85–0.95)	0.86 (0.73–1.00)	0.48 (0.39–0.59)	1.25 (1.,01–1.55)	0.97 (0.78–1.20)
Sardar et al. 2013 [64]	3	REM	OR	0.85 (0.74–0.99)	Not stated	0.37 (0.19–0.72)	0.90 (0.79–1.02)	0.84 (0.69–1.03)	0.42 (0.25–0.70)	1.17 (0.76–1.80)	Not stated
Senoo et al. [65]	3	REM	RR	0.45 (0.24–0.85)	Not stated	Not stated	Not stated	0.66 (0.29–1.47)	0.46 (0.18–1.16)	0.52 (0.25–1.08)	Not stated
Testa et al. 2012 [52]	3	FEM	OR	0.92 (0.83–1.02)	0.93 (0.83–1.05)	0.43 (0.34–0.55)	0.90 (0.84–0.96)	EC 0.98 (0.91–1.07)	Not stated	Not stated	1.03 (0.89–1.20)
NOACs vs. warfarin or ASA or VKA vs. NOACs/PAI (not included in analysis)											
Chatterje et al. 2013 [63]	6	REM	OR	Not stated	Not stated	Not stated	Not stated	Not stated	OR 0.49 (0.36–0.65)	Not stated	Not stated
Sardar et al. 2014 [32]	4	REM	OR	0.65 (0.48–0.87)	Not stated	Not stated	Not stated	0.82 (0.58–1.16)	Not stated	Not stated	Not stated
Agarwal et al. 2012	8	FEM	RR	1.44 (1.18–1.78)	Not stated	Not stated	Not stated	Not stated	Not stated	Not stated	Not stated

95% Confidence intervals are provided unless otherwise reported

*FEM* fixed effect model, *OR* odds ratio, *REM* random effect model, *RR* relative risk

##### Ischemic stroke

Nine SRs reported on ischemic stroke [28, 31, 47, 48, 50–54]. They included seven different studies. All SRs found a small advantage for NOACs with one reaching statistical significance: Miller et al. [50] who reported an RR 0.87 (95% CI 0.77–0.99) in favour of NOACs.

##### Haemorrhagic stroke

NOACs demonstrated a substantial advantage in reducing the risk of haemorrhagic strokes compared to warfarin across all nine SRs with seven underlying studies that examined this outcome [28, 31, 47, 48, 50, 52–54, 64]. The strongest pooled effect was reported by Sardar et al. [64] with an OR 0.37 (95% CI 0.19–0.72), and the smallest for the NOAC low dose subgroup by Jia et al. [54] (RR 0.33 (0.23–0.46)).

##### Mortality

**Fig. 3:**
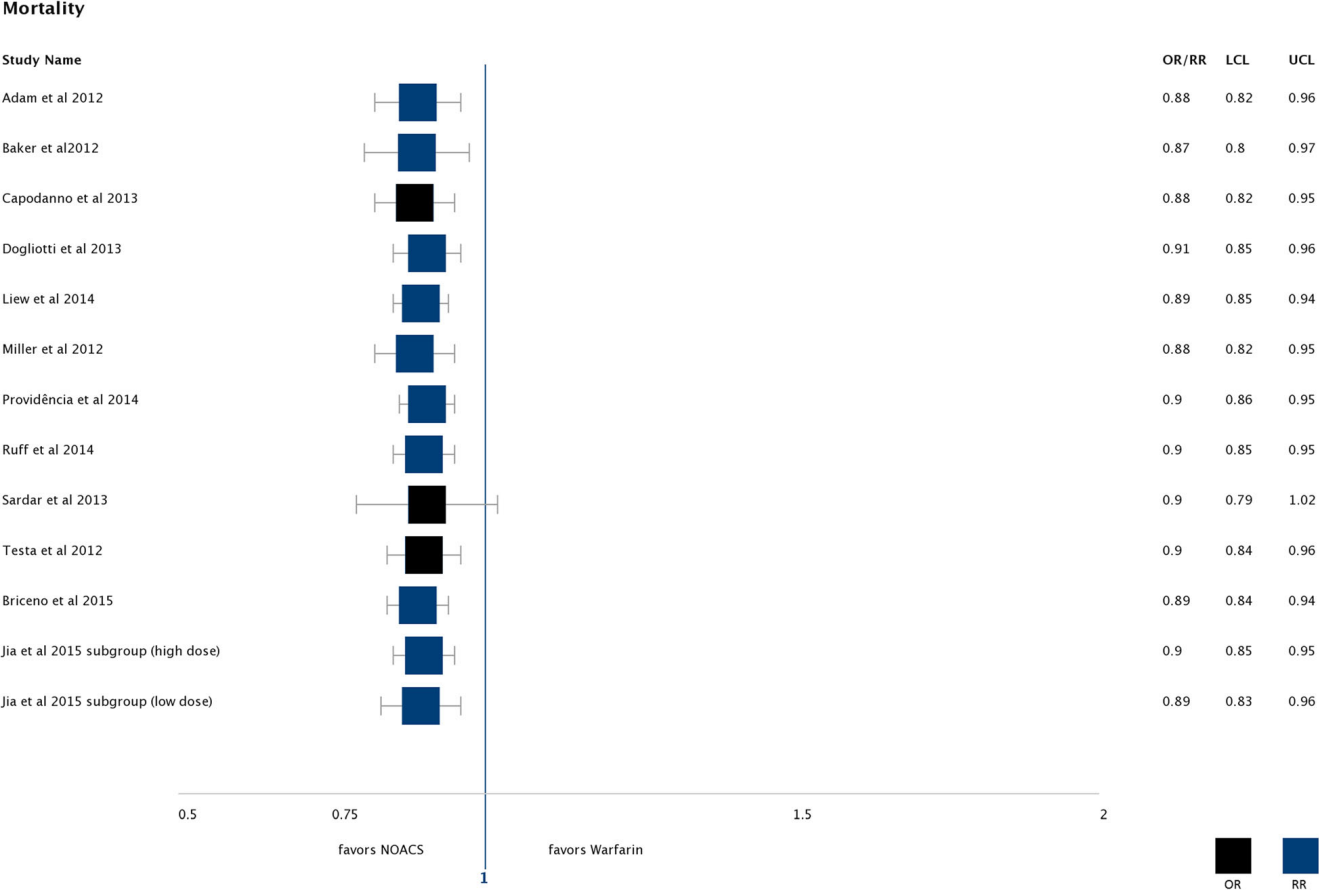
Mortality: Found effects favouring NOACs compared to warfarin

All the reviews showed a small benefit for NOACs compared to warfarin regarding mortality [28, 29, 31, 47–54, 64]. The effect estimates were highly consistent with a minimum RR of 0.874 (99% CI 0.803–0.974) [53] and a maximum RR of 0.91 (95% CI 0.85–0.96) [48] (see Fig. 3 and Table 2).

#### Safety outcomes

Safety outcomes were addressed by all included SRs except Adam et al. [31] who reported no safety outcomes for the subgroup of AF patients.

##### Major bleeding

**Fig. 4:**
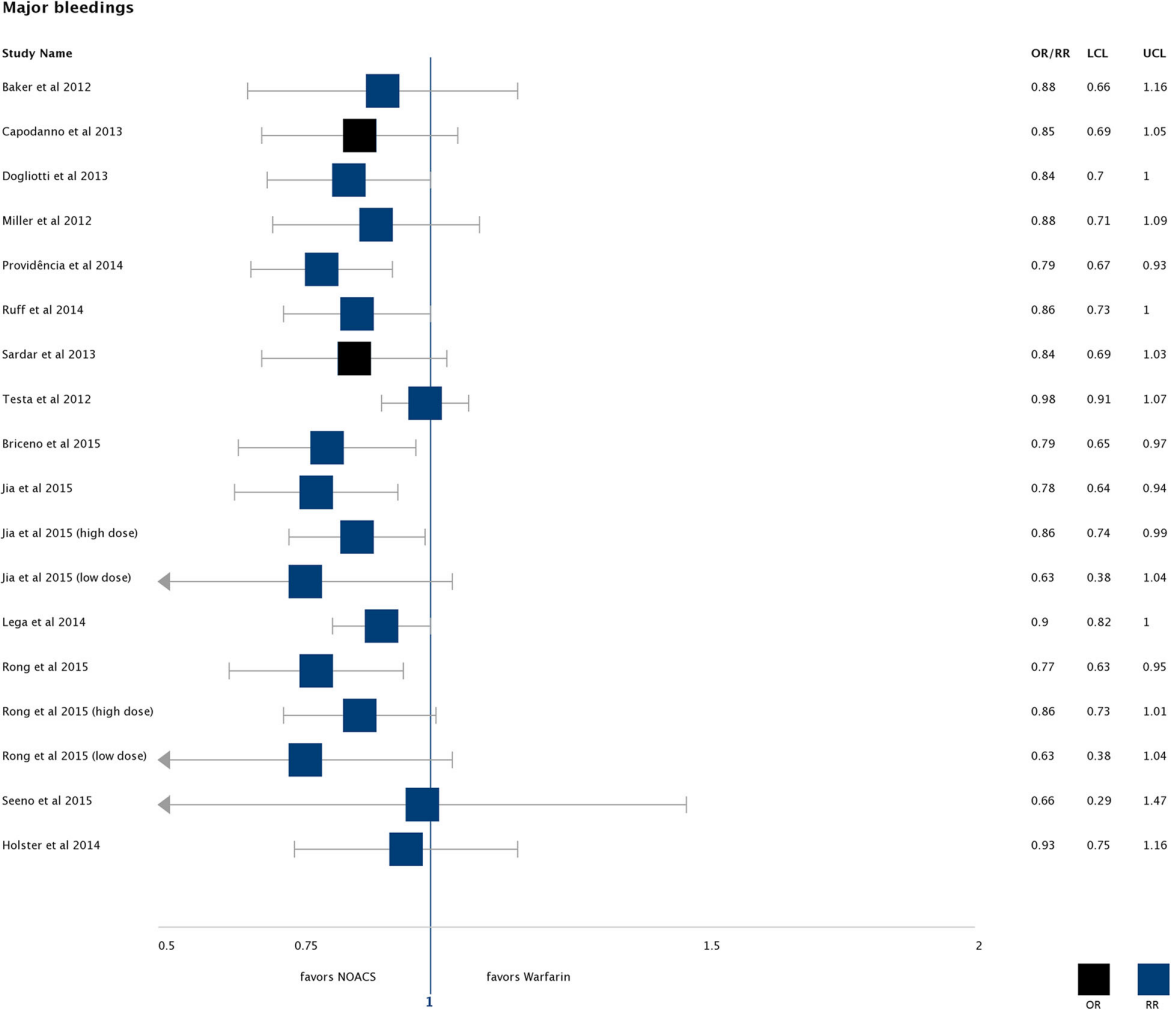
Major bleedings: Found effects favouring NOACs compared to warfarin

Fourteen SRs reported on major bleeding and addressed 12 single studies [28–30, 33, 47, 48, 50–54, 64–66]. There was a high heterogeneity for this outcome but the pooled effect estimates consistently favoured NOACs across all eight studies and were statistically significant in four studies [29, 51, 54, 66] (see Fig. 4).

##### Intracranial bleeding

**Fig. 5:**
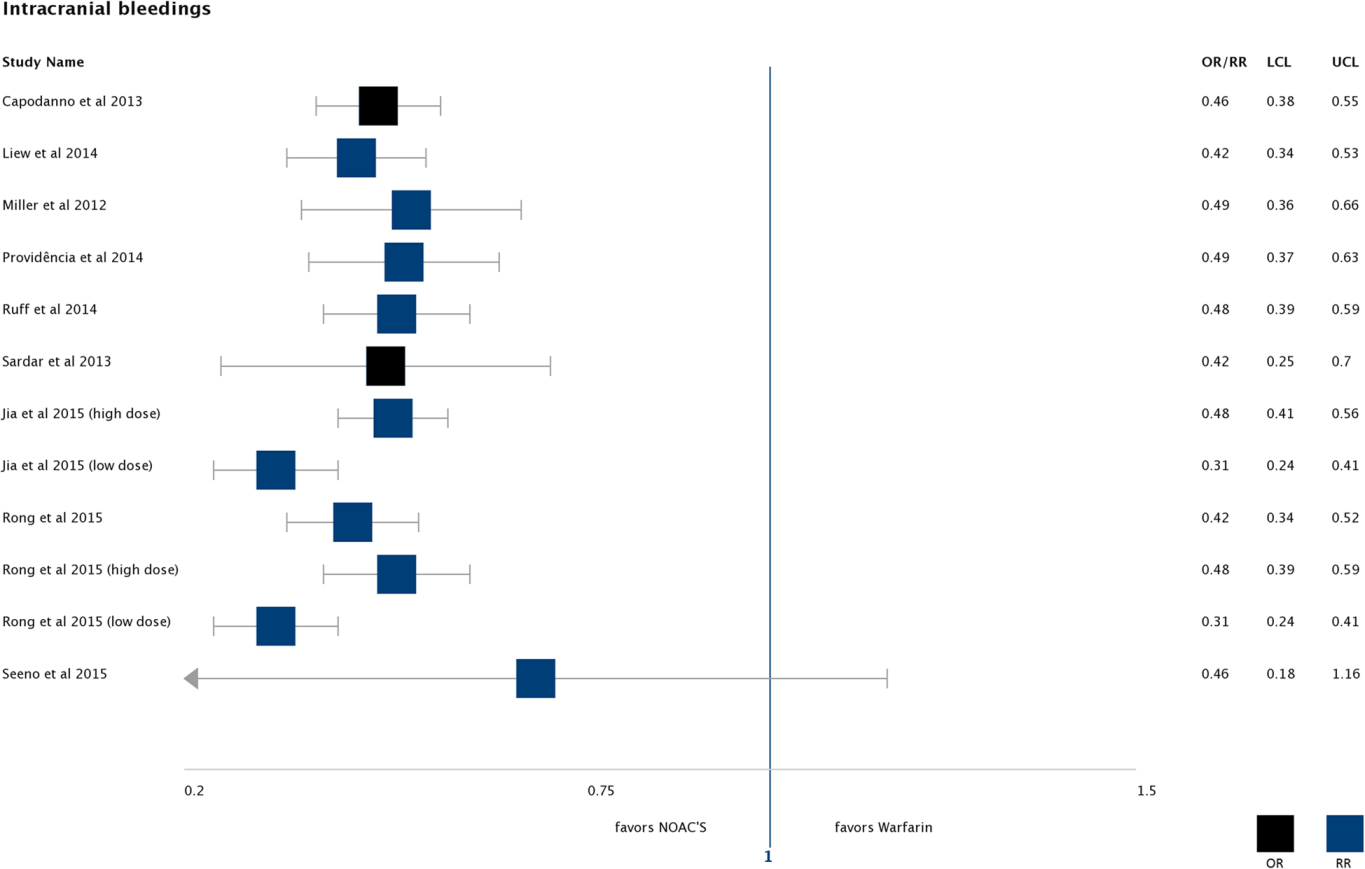
Intracranial bleedings: Found effects favouring NOACs compared to warfarin

A significant advantage for NOACs compared to warfarin was reported by all eight of nine reviews that reported on intracranial bleedings [28, 47, 49–51, 54, 64–66]. Seven single studies were included on this outcome in the SRs. All of these SRs reported a pooled effect estimate (OR or RR) of less than 0.5 in favour of NOACs. The effect was strongest in the two subgroups for low dose NOACs (see Fig. 5) [54, 66].

##### Gastrointestinal bleeding

Ten SRs reported on gastrointestinal bleeding adressing 11 single studies [28, 33, 47, 50, 51, 53, 54, 64–66]. All of these reported an increased risk for patients on NOACs compared to warfarin with except of one, though the effect estimates were quite variable and statistically significant in only three cases [28, 47, 54]. Senoo et al. reported a lower risk for NOACs for gastrointestinal bleeding but inlcuded only japanese patients. Jia et al. [54] reported only for high dose NOACs an increased risk for gastrointestinal bleeding.

##### Myocardial infarction

Six SRs reported on myocardial infarction and included six studies [28, 47, 50–52, 54]. Only Jia et al. [54] reported a higher risk for myocardial infarction when comparing low dose of NOACs to warfarin (RR 1.25 (1.04–1.50)). All other treatment effect estimates were close to a value of 1.0, inlcuding the comparison of high dose of NOACs vs. warfarin.

### Individual NOACs vs warfarin

Additionally, nine of the included SRs compared a single NOAC with VKA [26, 32, 35, 40, 42, 46, 67–69]. Two reviews exclusively included the comparison between dabigatran and VKA [35, 67]. Sharma et al. [26] performed a SR analysing patients aged 75 years or older and presented subgroup results for patients with AF. Six SRs performed a network meta-analysis (NMA) [40, 42, 43, 46, 67, 68] and four showed subgroup results for each NOAC [26, 32, 43, 69].

#### Stroke/SE

In the SRs presenting data on single NOACs, dabigatran was more effective for stroke prevention than VKA [35, 67]. This effect was also present in subgroup data for older patients ≥75 years [26, 32]. Sharma et al. [26] showed results for dabigatran 110 and 150 mg twice a day (BID) separately and the effect was only significant for higher doses of dabigatran. Sharma et al. [26] found a better effect in reducing stroke for apixaban compared to VKA in the subgroup of participants with AF. Rivaroxaban was as effective as warfarin regarding this outcome. Except for apixaban these results were based on one study for each drug only [26].

Schneeweiss et al. [69] performed a subgroup analysis for patients with CHADS_2_ score ≥ 3 with similar results with a significant reduction of stroke for dabigatran 150 mg BID and apixaban, but not for dabigatran 110 mg BID and rivaroxaban. Roskell et al. [67] reviewed the data from the RE-LY trial and showed significantly fewer ischemic stroke events with dabigatran 150 mg twice a day (RR 0.76, 95% CI 0.58–0.97) and fewer intracranial haemorrhage events (RR 0.52, 95% CI 0.32–0.84). Systemic embolism, mortality, extracranial haemorrhage and acute myocardial infarction did not significantly differ between the two treatment groups. Two NMA showed non-inferiority to warfarin for all single NOACs in the prevention of stroke [40, 42]. Two NMA showed an advantage for dabigatran [68] and two for apixaban [46, 68]. Three NMA [40, 67, 68] reported about ischemic stroke and only dabigatran 150 mg BID was superior to warfarin in one NMA [68]. Lin et al. [43] was the only NMA also including non-randomized trials but reported only an advantage for dabigatran 150 mg.

#### Mortality

Only edoxaban 30 mg showed a significant advantage compared to warfarin regarding mortality [26]. In the indirect comparisons**,** significant differences were found in two NMAs for apixaban which was associated with a lower mortality [43, 68], and in one NMA edoxaban 30 mg which was associated with higher mortality [68].

#### Bleeding

In the NMA of Verdecchia et al. [68] and Lin et al. [43] all investigated NOACs showed significantly fewer events with regard to intracranial bleeding, but this effect was not found in two other reviews [42, 67] and in the analysis of non-randomized trials for rivaroxaban in the SR of Lin et al. [43]. Warfarin showed fewer events of gastrointestinal bleeding than dabigatran 150 mg and rivaroxaban in the SR of Verdecchia et al. [68]. Edoxaban and apixaban were associated with less major bleeding in two NMAs [46, 68]. For all other comparisons no statistically significant differences were found.

#### Myocardial infarction

The NMA Lin et al. [43] found a reduced risk of myocardial infarction in the analysis of non-randomized trials for dabigatran (110 and 150 mg) compared to warfarin. There was no significant difference between single NOACs and VKA for myocardial infarction in two other NMA reporting this outcome [67, 68].

### Indirect comparison between NOACs

#### Effectiveness outcomes

No direct comparisons between the various NOACs are available. We identified ten SRs reporting a comparative effectiveness analysis via indirect comparisons between different kinds of NOACs (apixaban, dabigatran, rivaroxaban, edoxaban and ximelagatran) [40, 42, 43, 46, 52, 53, 64, 68–70]. Four NMAs reported ORs for the head to head adjusted indirect comparisons [46, 52, 64, 70]. Three NMAs [42, 43, 53] reported RR and Schneeweiss et al. [69] hazard ratios (HR). Two recent SRs (Dogliotti et al. [40] and Verdecchia et al. [68]) reported the surface under the cumulative ranking (SUCRA, %) [71]. They defined SUCRA as follows: “A simple numerical summary to supplement the graphical display of cumulative ranking is to estimate the surface under the cumulative ranking (SUCRA) line for each treatment; SUCRA would be 1 when a treatment is certain to be the best and 0 when a treatment is certain to be the worst.”

The single drugs are sorted out for each outcome according to a rating with a percentage of probability that each treatment is the best with respect to the next best treatment.

##### Stroke/SE

In Assiri et al. [42] edoxaban 30 mg was less effective in preventing stroke than other NOACs (dabigatran 150 mg, apixaban, edoxaban 60 mg, rivaroxaban) except dabigatran 110 mg. Dabigatran 150 mg was more effective than edoxaban 60 mg, rivaroxaban and dabigatran 110 mg. Two other indirect comparisons showed more stroke reduction with dabigatran than with rivaroxaban [53, 70]. In Harenberg et al. [70] and Lin et al. [43] compared both doses of dabigatran and the higher dose was more effective in reducing ischemic stroke/SE. For total stroke/systemic embolism the other head to head adjusted indirect comparisons in these two NMAs did not reveal any significant advantage for any one of the NOACs while both SUCRA-analyses ranked dabigatran 150 mg as best treatment with probabilities of 70% [40] and 97.2% [68], respectively.

##### Ischemic stroke

In both SUCRA-analyses dabigatran 150 mg was on the first position with 51 and 94.2%, respectively, and one SR found a lower risk for dabigatran than for rivaroxaban for ischemic stroke events [53].

##### Haemorragic stroke

In contrast, for the outcome haemorrhagic stroke only some of the indirect comparisons via ORs were significant, with inconsistent findings. One SR compared dabigatran 110 mg vs. rivaroxaban and another dabigatran 150 mg vs. rivaroxaban. Both found a lower risk of haemorrhagic stroke in favour of dabigatran ((OR 0.15, 95% CI 0.03–0.67) [64] and RR 0.454 (95% CI 0.210–0.983) [53], while another SR comparing dabigatran 150 mg with rivaroxaban did not show any significant differences (OR 0.85, 95% CI 0.65–1.11) [52]. Another comparison that showed a significant effect was apixaban vs. dabigatran 150 mg, with a lower risk of haemorrhagic stroke for dabigatran (OR 1.16, 95% CI 0.85–1.59) [52] but this result was not confirmed in another review that found no significant difference [53].

##### Mortality

No significant ORs of indirect comparison could be shown while SUCRA-analyses were inconsistent: Verdecchia et al. [68] ranked edoxaban first with a probability of 76.8%, and dabigatran 150 mg was given the second position with a probability of 65.1%, while Dogliotti et al. [40] ranked dabigatran 150 mg first with 31% probability but did not include edoxaban in the analysis.

#### Safety outcomes

##### Major bleeding

Verdecchia et al. [68] ranked edoxaban 30 mg first regarding the safety endpoint major bleeding (SUCRA 100%), followed by apixaban 5 mg (SUCRA 80.1%), edoxaban 60 mg (SUCRA 60.9%), dabigatran 110 mg (SUCRA 57.7%), dabigatran 150 mg (SUCRA 28.4%), adjusted dose warfarin (SUCRA 28.4%) and rivaroxaban 20 mg (SUCRA 9.2%). Dogliotti et al. [68] ranked quite differently in their SUCRA analysis (percentage representing the probability with the highest likelihood for that treatment for that rank): rank 1: control (87%); rank 2: ASA (44%); rank 3: apixaban (30%); rank 4: dabigatran 110 mg (26%); rank 5: dabigatran 150 mg (22%); rank 6: VKA (34%); rank 7: rivaroxaban (30%); and rank 8: ASA + clopidogrel (29%). Both rankings showed the same order for apixaban, dabigatran and rivaroxaban. This was in line with the results of the indirect comparisons of Sardar et al. [64]. They found a significant difference between apixaban vs. dabigatran 150 mg and apixaban vs. rivaroxaban both in favour of apixaban (OR 0.19, 95% CI 0.13–0.28 and OR 0.19, 95% CI 0.14–0.28). In addition there was a significantly lower rate of major bleeding favouring dabigatran 110 mg in comparison with rivaroxaban (OR 0.68, 95% CI 0.46–0.99) [64]. These results were reproduced in five other indirect comparisons showing significantly less major bleeding for apixaban compared to dabigatran and compared to rivaroxaban [43, 46, 53, 69, 70]. Additionally, Cameron et al. [46] showed that edoxaban 30 mg was less effective than other NOACs and also showed less major bleeding compared to all other NOACs. Only the review of Assiri et al. [42] showed no significant difference for any comparison between apixaban, dabigatran, rivaroxaban and edoxaban. Lin et al. [43] showed less major bleedings with the lower dose of dabigtran (110 mg) compared to higher dose and to rivaroxaban.

##### Intracranial bleeding

Sardar et al. [64] reported a significantly lower rate for dabigatran 110 mg compared with rivaroxaban (OR 0.27, 95% CI 0.10–0.73) which is in line with the findings by Verdecchia et al. [68] who ranked dabigatran first in their SUCRA analysis with a probability of 88.7% and rivaroxaban second to last with a probability of 18.9%. Lin et al. [43] found a lower risk for apixaban compared to dabigatran and in their analysis of non-randomized trials, rivaroxaban was associated with a higher risk of intracranial bleeding than dabigatran 150 mg. Two other indirect comparisons found no significant advantage for any of the single NOACs for intracranial bleeding [42, 70].

##### Gastrointestinal bleeding

Verdecchia et al. [68] reported the following cumulative ranking: edoxaban 30 mg (SUCRA 90.3%), apixaban 5 mg (SUCRA 78.9%), adjusted-dose warfarin (SUCRA 64.6%), dabigatran 110 mg (SUCRA 53.0%), edoxaban 60 mg (SUCRA 34.6%), dabigatran 150 mg (SUCRA 14.7%) and rivaroxaban 20 mg (SUCRA 5.4%). Sardar et al. [64] reported nonsignificant differences in the indirect comparison between apixaban and dabigatran for both doses while Baker et al. [53] found a lower incidence for gastrointestinal bleedings for apixaban compared to rivaroxaban and compared to dabigatran. Lin et al. [43] found no significant differences.

##### Myocardial infarction

Verdecchia et al. [68] reported a safety ranking for myocardial infarction in the following order (percentage of SUCRA): rivaroxaban 20 mg (90.3%), apixaban 5 mg (77.7%), edoxaban 60 mg (68.6%), adjusted dose warfarin (56.8%), edoxaban 30 mg (23.9%), dabigatran 150 mg (17.9%), dabigatran 110 mg (14.7%). The disadvantage of dabigatran regarding MI in both doses was in line with the ORs of the indirect comparisons by Testa et al. [52] and Harenberg et al. [70]. Testa et al. [52] found significant differences in the comparison of apixaban vs. dabigatran 110 mg and apixaban vs. dabigatran 150 mg favouring apixaban (OR 0.6, 95% CI 0.4–0.9 and OR 0.6, 95% CI 0.4–0.96, respectively). The comparison of the two doses of dabigatran with rivaroxaban revealed similar results favouring rivaroxaban (OR 1.7, 95% CI 1.12–2.6 and OR 1.76, 95% CI 1.1–2.6, respectively) [52] with similar results found by Harenberg et al. (OR 1.64, 95% CI 1.09–2.45) for dabigatran 110 mg vs. rivaroxaban and for dabigatran 150 mg vs. rivaroxaban (OR 1.61, 95% CI 1.09–2.41). Lin et al. found no significant differences.

### Quality appraisal of included SR and MA

Table 3 displays the results of each item of the AMSTAR tool for each SR, where positive answers are related to a low risk of bias. Overall, no SR met all quality criteria suggesting a moderate quality of evidence for most SRs.

**Table 3:** Quality appraisal of included systematic reviews

Reference	1. Was an ‘a priori’ design provided?	2. Was there duplicate study selection and data extraction?	3. Was a comprehensive literature search performed?	4. Was the status of publication (i.e. grey literature) used as an inclusion criterion?	5. Was a list of studies (included and excluded) provided?	6. Were the characteristics of the included studies provided?	7. Was the scientific quality of the included studies assessed and documented?	8. Was the scientific quality of the included studies used appropriately in formulating conclusions?	9. Were the methods used to combine the findings of studies appropriate?	10. Was the likelihood of publication bias assessed?	11. Was the conflict of interest stated?
Adam et al. 2012 [31]	yes	yes	yes	no	yes	yes	yes	yes	yes	unclear	yes
Agarwal et al. 2012 [27]	unclear	no	yes	no	no	yes	yes	yes	yes	yes	no
Aguilar et al. 2005 [34]	yes	yes	yes	unclear	yes	yes	yes	yes	yes	no	yes
Aguilar et al. 2007 [44]	yes	yes	yes	unclear	yes	yes	yes	yes	yes	no	yes
Andersen et al. 2008 [37]	yes	yes	yes	no	yes	yes	yes	yes	NA	yes	yes
Assiri et al. 2013 [42]	yes	unclear	yes	no	no	yes	unclear	unclear	yes	no	yes
Baker et al. 2012 [53]	yes	yes	yes	yes	no	yes	yes	yes	yes	yes	yes
Briceno et al. 2015 [29]	yes	unclear	yes	no	no	yes	no	unclear	yes	yes	yes
Cameron et al. 2014 [46]	yes	yes	yes	yes	yes	yes	no	no	yes	no	yes
Capodanno et al. 2013 [47]	yes	yes	yes	no	no	yes	no	no	yes	yes	no
Chatterje et al. 2013 [63]	yes	yes	yes	no	no	yes	yes	yes	yes	yes	yes
Coleman et al. 2012 [35]	yes	yes	yes	no	yes	yes	yes	yes	yes	yes	no
Cooper et al. 2006 [41]	yes	no	yes	no	no	yes	no	unclear	no	no	no
Dogliotti et al. 2013 [48]	yes	yes	yes	no	no	yes	no	no	yes	yes	yes
Dogliotti et al. 2014 [40]	yes	yes	yes	no	no	yes	no	yes	no	no	yes
Harenberg et al. 2012 [70]	yes	unclear	yes	no	no	yes	no	unclear	yes	no	yes
Hart et al. 1999 [4]	yes	yes	yes	no	no	yes	no	unclear	yes	no	no
Hart et al. 2007 [39]	yes	yes	yes	yes	yes	yes	yes	yes	yes	no	yes
Holster et al. 2013 [33]	yes	yes	yes	unclear	no	no	yes	yes	yes	yes	no
Jia et al. 2014 [54]	yes	unclear	yes	yes	yes	no	no	unclear	yes	no	no
Lega et al. 2014 [30]	yes	yes	yes	yes	yes	no	yes	yes	yes	no	yes
Liew et al. 2014 [49]	yes	yes	yes	unclear	no	no	yes	yes	yes	no	yes
Lin et al. 2015 [43]	yes	yes	yes	yes	yes	yes	yes	no	no	yes	yes
Lip et al. 2006 [36]	yes	no	yes	no	no	yes	no	unclear	yes	yes	yes
Miller et al. 2012 [50]	yes	yes	yes	yes	no	yes	no	no	yes	no	no
Providência et al. 2014 [51]	yes	yes	yes	yes	unclear	yes	unclear	unclear	yes	no	yes
Rong et al. 2015 [66]	yes	unclear	yes	yes	no	yes	yes	yes	yes	no	yes
Roskell et al. 2010 [67]	NA	yes	yes	no	no	no	yes	yes	yes	no	yes
Ruff et al. 2014 [28]	yes	unclear	no	no	no	yes	no	unclear	yes	unclear	yes
Sardar et al. 2013 [64]	yes	yes	yes	yes	no	yes	yes	yes	yes	yes	yes
Sardar et al. 2014 [32]	yes	yes	yes	no	no	yes	yes	unclear	yes	yes	yes
Schneeweiss et al. 2012 [69]	yes	unclear	yes	no	no	no	no	unclear	yes	no	yes
Segal et al. 2000 [38]	yes	yes	yes	no	yes	yes	yes	yes	yes	no	yes
Senoo et al. 2015 [65]	yes	unclear	yes	yes	no	yes	yes	yes	yes	no	yes
Sharma et al. 2015 [26]	yes	yes	yes	yes	no	yes	yes	no	yes	yes	yes
Taylor et al.2001 [45]	yes	unclear	yes	yes	no	yes	unclear	yes	yes	yes	yes
Testa et al. 2012 [52]	yes	unclear	yes	no	no	unclear	yes	unclear	yes	no	no
Verdecchia et al. 2015 [68]	yes	yes	yes	unclear	no	yes	no	unclear	yes	no	yes

*NA* not applicable

We obtained the information on the quality of the single studies that were relevant for the development of the recommendations. All studies were judged with low risk of bias [55–57, 60].

## Recommendations

For older patients with AF, we found a considerable advantage for NOACs compared to VKAs regarding haemorrhagic strokes/intracranial haemorrhages and a small benefit regarding mortality. From these results we were able to develop a weak recommendation to switch from a VKA to a NOAC in older patients with atrial fibrillation (see Additional file 7: Table S7). We restricted the recommendation to patients with a low time in therapeutic range (TTR) below 55% because this was the lower limit of TTR in the approval studies of NOACs and it remains unclear to what extent the advantages apply to patients with high time in therapeutic range [72]. The quality of evidence for this recommendation is moderate. The quality of the evidence was downgraded because of indirectness of the results as there is no trial that evaluated the effect of switching from vitamin K antagonists to a novel oral anticoagulant. We considered three guidelines as additional articles of interest [8, 73, 74]. They recommend a conventional VKA for patients with severe renal impairment and therefore we excluded patients with severe renal impairment from our recommendation.

The recommendation was developed according to our methods for the compilation of SRs. Meetings with the team of researchers were held to discuss and agree on the recommendation reflecting the strength and quality of evidence according to the results of our SR. The recommendation was subsequently reviewed and confirmed by the Evidence based Medicine Guidelines Editorial board of Duodecim Medical Publication Ltd. (Finland) and will be implemented in the electronic decision support tool PRIMA-eDS.

## Discussion

We performed a review of systematic reviews to investigate the effectiveness and safety of vitamin K antagonists and new oral anticoagulants in older patients with atrial fibrillation. The primary aim of this review was to inform stop recommendations regarding medication use in older people to reduce inappropriate polypharmacy. A general stop recommendation on use of anticoagulants in older people with AF was not justified, because the evidence identified in our review showed a clear benefit for VKA compared to PAI as well as to placebo for the prevention of strokes outweighing the risk of major bleeding. These findings are consistent with existing guideline recommendations [8, 74, 75].

NOACs were associated with a reduced risk of haemorrhagic strokes and intracranial bleedings compared to VKA, though potentially some increase in gastrointestinal bleeding. A small reduction in risk of mortality in favour of NOACs was also observed. Overall, from all data currently available, there appears to be at least equipoise between VKA and NOAC regarding benefit, and a small advantage for NOACs regarding harm, but effect sizes are small (reduction of intracranial haemorrhage for rivaroxaban with a NNH estimate of about 500 calculated from the ROCKET-AF-trial, reduction of major bleeding for apixaban with a NNH estimate of about 100 calculated from the ARISTOTLE-trial, and a reduction of major bleeding for dabigatran (110 mg) with a NNH estimate of about 150 calculated from the RE-LY-trial). We therefore recommend considering the use of NOACs in patients aged 65 years or older with AF as well as considering a switch from a VKA to a NOAC, particularly if the time in therapeutic range is low with VKA. It must be emphasized, though, that the latter recommendation is based on indirect evidence, because a trial investigating the effects of switching from VKA to NOAKs does not exist.

In the existing guidelines of NICE and the AHA/ACC, NOACs are recommended equally to warfarin [8, 74]. However, the AHA/ACC guideline recommendations for dabigatran, rivaroxaban and apixaban are associated with a lower level of evidence (B = moderate quality of evidence, further research is likely to have an important impact on our confidence in the estimate of effect and may change the estimate) than for warfarin (A = high quality of evidence, further research is very unlikely to change our confidence in the estimate of the effect) [74]. In contrast, the ESC guidelines recommend a NOAC in the majority of patients [6]. A prescription of NOACs also appears to be recommendable for patients with labile INR.

Our findings are in line with the recommendations in the ESC guidelines. However, there are still concerns as to whether these recommendations are equally applicable to the very elderly with multiple comorbidities [6]. We assessed the available patient characteristics for all included SRs. Participants in the studies were mostly aged over 65 years, due to the epidemiological characteristics of the disease, but the studies provided no data on patient frailty, cognitive status or polypharmacy. Thus, the evidence for older people who are affected by multiple comorbidities is less clear.

The NICE and the AHA/ACC/HRS guidelines recommend conventional therapy with VKA in patients with severe renal impairment [8, 74], a common condition in older people. This applies especially to avoidance of dabigatran which is eliminated mainly by renal excretion and for which a number of cases of major bleeding, mostly in older adults with severely impaired renal function, have been reported [76]. In a secondary analysis of the RE-LY trial data Eikelboom et al. [77] found a significant treatment-by-age-effect, by which patients aged ≥75 years had a higher risk of major bleeding with dabigatran than with warfarin, whereas in younger patients the association was reversed [55, 77]. Several case reports have suggested that the risk of gastrointestinal bleeding may also be higher with dabigatran, mostly associated with impaired renal function [78]; this concurs with the trend we observed in our analysis for NOACs in general. Further research on this issue is needed to determine whether this effect is restricted to particular NOACs or to specific patient groups.

Head to head comparisons of individual NOACS are lacking, hence the only data on these were indirect comparisons. Overall, there seemed to be a trend for better safety with apixaban and better efficacy with dabigatran regarding risk of strokes, but a higher incidence of myocardial infarction associated with dabigatran. However, the lack of direct evidence makes it impossible to definitively recommend one NOAC over another. In the RE-LY trial dabigatran at a dose of 150 mg twice a day was associated with a higher risk of myocardial infarction compared to warfarin [55]. This finding was confirmed in an MA by Uchino et al. [79] that included data from trials for all indications for anticoagulation. In our SR we found no higher incidence of MI for NOACs as a class of drugs. In a Danish cohort study dabigatran 110 and 150 mg twice a day showed both lower incidence of MI than warfarin with low overall incidence of MI [80]. The rates of stroke and major bleeding were similar between dabigatran and warfarin in this cohort and dabigatran was associated with less intracranial bleeding and a lower mortality. We found similar results in our SR.

Our recommendation to switch from a VKA to NOACs currently relates to AF patients with a low TTR only. This is to reflect the range of TTR in the relevant trials, of 55–68% [55–57, 60]. It is not clear if the advantages of NOACs would also apply to patients with a higher TTR, as the treatment effect of warfarin is associated with the time spent in therapeutic range [81]. However, patients on warfarin most likely spend a significant proportion of time outside of the TTR, as suggested by Van Walraven et al. [82] who, from a meta-analysis of 50,208 patients, reported an average TTR of 63.6%, with a tendency of a lower TTR in community practices than in clinical trials.

Further advantages of NOACs are that they do not require patients to undergo regular blood tests and they have fewer food and drug interactions than VKAs. However, at present only one specific antidote for NOACs is. Idarucizumab is a monoclonal antibody that reverses the anticoagulation effects of dabigatran [83], approved in 2015 by the Food & Drug Administration (FDA) and European Medicines Agency (EMA) as the first reversal agent for new oral anticoagulants. Andexanet alfa is a further specific antidote for factor Xa inhibitors such as apixaban and rivaroxaban, and its approval by the FDA is expected in 2017 [84]. The effect of VKA can be reversed by administration of Vitamin K but the reversal is tardy and not well controllable.

This SR has limitations. We performed a systematic review of systematic reviews, and therefore synthesised the evidence of already synthesised evidence, with some associated loss of information. A further key limitation is that our methodology allowed SRs based on the same, or nearly the same, set of only a few underlying individual studies to be jointly included in our synthesis. This resulted in a considerable degree of evidence overlap between the included SRs. However, we summarised the results from all SRs regardless of the degree of overlap: analysis models and definitions of outcomes varied between SRs, even when the set of included studies was identical, providing what can be considered to be replications by different research teams addressing the same research question. Nonetheless, it is important when considering our findings, to bear in mind that the studies (SRs) being synthesised by no means represent independent pieces of evidence.

In the discussion of the methodology of umbrella SRs like ours, some authors prefer to select only one SR for reporting if there is relevant overlap of studies in several existing SRs [85]. With regard to which systematic review might be chosen Cooper and Koenka [86] suggest selecting the synthesis that (1) provides the most complete description, (2) is most recent, (3) contains the most evidence, (4) is methodologically most rigorous, and/or (5) is published. We feel that in our case this approach would result in excluding several systematic reviews that offer relevant additional information. Although the quality of the SRs we included in our umbrella-SR was fairly good, none of the SRs fulfilled all AMSTAR quality criteria. We therefore don’t consider it justified to select a single SR. Instead, we report transparently which individual studies were included in the different SRs and which outcomes were addressed [86].

Although all individual studies were rated low risk of bias, the number of unique studies addressing each drug comparison were fairly small, making the sufficiency of the available evidence debatable.

One of our exclusion criteria related to patient age, which may have resulted in some relevant SRs being excluded where this data was not reported. This risk was minimised however, through the examination of the full texts of references where these data were unclear in the abstracts. We also checked the reference lists of all included studies to identify further eligible SRs. Accordingly there were no statistically significant differences of effects between the SRs. The frequently small differences in effects can be explained by rounding errors or heterogeneity of the definition of outcomes, used effect measures, used statistical models and other factors.

With this study, we provide a thorough overview about anticoagulation in older people with AF including several comparisons and evidence for all relevant comparisons. Because of our methodology of a review of systematic reviews we were only able to do a qualitative synthesis and a loss of information cannot be ruled out. We tried to avoid the loss of information by carefully assessing all available data on all outcomes and in addition on patient characteristics. We focused on people aged 65 years and older but data on frailty, cognitive status and polypharmacy were very limited. NOACs are a very actual topic and we were able to provide recent data. For example, the very recent studies on edoxaban so far have not been considered universally in guidelines.

The recommendations based on this review will be implemented in an electronic decision support tool to reduce inappropriate polypharmacy in older adults. This tool will be tested in a large multicentre randomized trial with over 3600 patients in Germany, Austria, Italy and the UK.

## Conclusions

Anticoagulation with vitamin K antagonists in older people with atrial fibrillation is beneficial in comparison to PAI and placebo. Current best evidence suggests that new oral anticoagulants substantially reduce the risks of haemorrhagic strokes and intracranial bleedings relative to VKAs, and should be considered especially in older people with low TTR and labile INR. However, the applicability of these results to frail, cognitively impaired or multimorbid older people is unclear.

## Additional files

Additional file 1: Search strings. (DOCX 130 kb)

Additional file 2: Table S1. Patient characteristics of the included SRs: Summary of study characteristics. (DOCX 28 kb)

Additional file 3: Table S2. Dates and databases of Systematic Review searches. (DOCX 91 kb)

Additional file 4: Table S3. Summary of study findings for the comparison of VKA vs. placebo. **Table S4.** Summary of study findings for the comparison of VKA vs. antiplatelets. (DOCX 28 kb)

Additional file 5: Table S5. Heterogenity. (DOCX 52 kb)

Additional file 6: Table S6. Eventrates NOACs and VKA. (DOCX 24 kb)

Additional file 7: Table S7. Recommendation. (DOCX 16 kb)

## Abbreviations

ACC: American College of Cardiology
AHA: American Heart Association
AMSTAR: A Measurement Tool to Assess Systematic Reviews
ARISTOTLE: Apixaban for Reduction in Stroke and Other Thromboembolic Events in Atrial Fibrillation
ASA: Acetylsalicylic acid
CDSR: Cochrane Database of Systematic Reviews
DARE: Database of Abstracts of Reviews of Effects
EAFT: European Atrial Fibrillation Trial
EC: Extracranial
EMA: European Medicines Agency
EMBASE: Excerpta Medica Database
ENGAGE AF-TIMI 48: Effective Anticoagulation With Factor Xa Next Generation in Atrial Fibrillation
ESC: European Society of Cardiology
FDA: Food and Drug Administration
FEM: Fixed effect model
GRADE: Grading of Recommendations Assessment, Development and Evaluation
HS: Haemorrhagic stroke
HTA: Health Technology Assessment
INR: International Normalized Ratio
IPA: International Pharmaceutical Abstracts
IS: Ischemic stroke
J-ROCKET AF: Japan-Rivaroxaban Once Daily Oral Direct Factor Xa Inhibition Compared with Vitamin K Antagonism for Prevention of Stroke and Embolism Trial in Atrial Fibrillation
MA: Meta-analysis
MI: Myocardial infarction
MTM: Multiple-Treatments Meta-analysis
NICE: National Institute for Health and Care Excellence
NMA: Network meta-analysis
NOAC: New oral anticoagulant
OR: Odds ratio
PICOS: Patient, Intervention, Comparator, Outcome, Setting
PRIMA-eDS: Polypharmacy in chronic diseases: Reduction of Inappropriate Medication and Adverse drug events in elderly populations by electronic Decision Support
PRISMA: Preferred Reporting Items for Systematic Reviews and Meta-Analyses
RE-LY: Randomized Evaluation of Long Term Anticoagulant Therapy
REM: Random effect model
ROCKET AF: Rivaroxaban Once Daily Oral Direct Factor Xa Inhibition Compared with Vitamin K Antagonism for Prevention of Stroke and Embolism Trial in Atrial Fibrillation
RR: Risk Ratio
RRR: Relative risk reduction
SE: Systemic embolism
SPORTIF: Stroke Prevention using Oral Thrombin Inhibitor in atrial Fibrillation
SR: Systematic review
SUCRA: Surface under the cumulative ranking
TTR: Time in therapeutic range
VKA: Vitamin K antagonist
