# Correction to: Effectiveness and safety of vitamin K antagonists and new anticoagulants in the prevention of thromboembolism in atrial fibrillation in older adults – a systematic review of reviews and the development of recommendations to reduce inappropriate prescribing

**DOI:** 10.1186/s12877-017-0663-5

**Published:** 2018-01-16

**Authors:** Christina Sommerauer, Lisa Schlender, Marc Krause, Sabine Weißbach, Anja Rieckert, Yolanda V. Martinez, David Reeves, Anna Renom-Guiteras, Ilkka Kunnamo, Andreas Sönnichsen

**Affiliations:** 1 Institute of General Practice and Family Medicine, University of Witten/ Herdecke, Alfred-Herrhausen-Straße 50, 58448 Witten, Germany; 2 NIHR School for Primary Care Research, Manchester Academic Health Science Centre, University of Manchester, Manchester, UK; 3 Department of Geriatrics, University Hospital Parc de Salut Mar, Barcelona, Spain; 4 Duodecim Medical Publications Ltd, Helsinki, Finland

## Correction

After publication of the original article [1] it was found that author Marc Krause’s name had been spelt incorrectly.

In the original article it is presented as Mark Krause, rather than Marc Krause. The revised spelling has been included in the author list for this Correction.
