# Supplementary material for: Effectiveness and safety of vitamin K antagonists and new anticoagulants in the prevention of thromboembolism in atrial fibrillation in older adults – a systematic review of reviews and the development of recommendations to reduce inappropriate prescribing
Source: BMC Geriatr. 2017 Oct 16;17(Suppl 1):223. doi: 10.1186/s12877-017-0573-6 (PMC5647558; doi:10.1186/s12877-017-0573-6)
Supplement: Supplementary file 1 — Search strings. (DOCX 130 kb) [file 12877_2017_573_MOESM1_ESM.docx]

**Search 1 and 2: VKA/NOACs for the management of atrial fibrillation**

**Databases:**

**EBM Reviews - Cochrane Database of Systematic Reviews [search 1]**

**EBM Reviews - Database of Abstracts of Reviews of Effects [search 1]**

**Ovid MEDLINE(R) [search 2]**

**Ovid MEDLINE(R) In-Process & Other Non-Indexed Citations [search 2]**

**Embase [search 2]**

**EBM Reviews - Health Technology Assessment [search 2]**

**International Pharmaceutical Abstracts [search 2]**

| [**# ▲**](http://ovidsp.uk.ovid.com/sp-3.8.1a/ovidweb.cgi?&S=DCDLPDGALHHFOEDMFNOKEBEGNLKJAA00&Sort+Sets=descending) |  |  |
| --- | --- | --- |
| **Population** | | |
|  | geriatrics.mp. or exp geriatrics/ |  |
|  | geriatric patient.mp. |  |
|  | geriatric*.mp. |  |
|  | (elder$ or geriatric$).ab,ti. |  |
|  | elder*.mp. |  |
|  | frail elderly.mp. or exp frail elderly/ |  |
|  | aged.mp. or exp Aged/ |  |
|  | old*.mp. |  |
|  | old* adult*.mp. |  |
|  | old* people*.mp. |  |
|  | >65.mp. |  |
|  | over 65.mp. |  |
|  | or/1-12 |  |
| **Condition** | | |
|  | Atrial Fibrillation.mp. or exp Atrial Fibrillation/ |  |
|  | Thromboembolism.mp. or exp Thromboembolism/ |  |
|  | or/14-15 |  |
| **Intervention** | | |
|  | Oral anticoagulants.mp. |  |
|  | vitamin k antagonist*.mp. |  |
|  | antivitamin k.mp. |  |
|  | dicoumarol.mp. |  |
|  | phenindione.mp. |  |
|  | warfarin.mp. |  |
|  | phenprocoumon.mp. |  |
|  | acenocoumarol.mp. |  |
|  | ethyl biscoumacetate.mp. |  |
|  | clorindione.mp. |  |
|  | diphenadione.mp. |  |
|  | tioclomarol.mp. |  |
|  | fluindione.mp. |  |
|  | Antithrombins.mp. or exp Antithrombins/ |  |
|  | new anticoagulants.mp. |  |
|  | Direct thrombin inhibitors.mp. |  |
|  | desirudin.mp. |  |
|  | lepirudin.mp. |  |
|  | argatroban.mp. |  |
|  | melagatran.mp. |  |
|  | ximelagatran.mp. |  |
|  | bivalirudin.mp. |  |
|  | dabigatran etexilate.mp. |  |
|  | dabigatran.mp. |  |
|  | Direct factor Xa inhibitors.mp. |  |
|  | rivaroxaban.mp. |  |
|  | apixaban.mp. |  |
|  | or/17-43 |  |
| **Outcome** | | |
|  | mortality.mp. or exp mortality/ |  |
|  | quality of life.mp. or exp quality of life/ |  |
|  | QOL.mp. |  |
|  | cardiovascular event.mp. |  |
|  | myocardial infarction.mp. |  |
|  | stroke.mp. |  |
|  | Ischemic Attack, Transient.mp. |  |
|  | [exp Ischemic Attack, Transient/] |  |
|  | hospitalization.mp. or exp hospitalization/ |  |
|  | hospitalisation.mp. or exp hospitalisation/ |  |
|  | life expectancy.mp. |  |
|  | cognitive impairment.mp. |  |
|  | cognitive status.mp. |  |
|  | functional status.mp. |  |
|  | functional impairment.mp. |  |
|  | renal failure.mp. |  |
|  | renal insufficiency.mp. or exp renal insufficiency/ |  |
|  | adverse drug event.mp. |  |
|  | adverse effects.mp. or exp adverse effects/ |  |
|  | drug toxicity.mp. or exp drug toxicity/ |  |
|  | safety.mp. |  |
|  | patient safety.mp. or exp patient safety/ |  |
|  | falls.mp. |  |
|  | delirium.mp. or exp delirium/ |  |
|  | or/45-68 |  |
| **Limits, Study designs** | | |
|  | (systematic review.ti. or meta-analysis.pt. or meta-analysis.ti. or systematic literature review.ti. or (systematic review.ti,ab. and review.pt.) or consensus development conference.pt. or practice guideline.pt. or cochrane database of systematic reviews.jn. or acp journal club.jn. or health technology assessment winchester england.jn. or evidence report technology assessment summary.jn. or drug class reviews.ti. or (clinical guideline and management).tw. or ((evidence based.ti. or evidence-based medicine.sh. or best practice*.ti. or evidence synthesis.ti,ab.) and (((review.pt. or diseases category.mp. or behaviour.sh.) and behavior mechanisms.mp.) or therapeutics.sh. or evaluation studies.pt. or validation studies.pt. or guideline.pt. or pmcbook.mp.)) or (((systematic or systematically).tw. or critical.ti,ab. or study selection.tw. or ((predetermined or inclusion) and criteri*).tw. or exclusion criteri*.tw. or main outcome measures.tw. or standard of care.tw. or standards of care.tw.) and ((survey or surveys).ti,ab. or overview*.tw. or review.ti,ab. or reviews.ti,ab. or search*.tw. or handsearch.tw. or analysis.ti,ab. or critique.ti,ab. or appraisal.tw. or (reduction.tw. and (risk.sh. or risk.tw.) and (death or recurrence).mp.)) and ((literature or articles or publications or publication or bibliography or bibliographies or published).ti,ab. or unpublished.tw. or citation.tw. or citations.tw. or database.ti,ab. or internet.ti,ab. or textbooks.ti,ab. or references.tw. or scales.tw. or papers.tw. or datasets.tw. or trials.ti,ab. or meta-analy*.tw. or (clinical and studies).ti,ab. or treatment outcome.sh. or treatment outcome.tw. or pmcbook.mp.))) not (letter or newspaper article or comment).pt. |  |
