# Supplementary material for: Effectiveness and safety of vitamin K antagonists and new anticoagulants in the prevention of thromboembolism in atrial fibrillation in older adults – a systematic review of reviews and the development of recommendations to reduce inappropriate prescribing
Source: BMC Geriatr. 2017 Oct 16;17(Suppl 1):223. doi: 10.1186/s12877-017-0573-6 (PMC5647558; doi:10.1186/s12877-017-0573-6)
Supplement: Supplementary file 2 — Patient characteristics of the included SRs: Summary of study characteristics. (DOCX 28 kb) [file 12877_2017_573_MOESM2_ESM.docx]

Additional file 2: Table S1 - Patient characteristics of the included SRs

| **Authors/ publication year** | **Inclusion criteria** | **Exclusion**  **criteria** | **Male sex**  **(%)** | **Mean age**  **(years)** | **Comorbidities** | **CHADS2 Score** | **TTR**  **(%)** |
| --- | --- | --- | --- | --- | --- | --- | --- |
| Adam et al. 2012 [25] |  | ximelagatran | 60.3-64.7 | ≥70 | Not reported | 2.1-3.5 | Median 64  (55-65) |
| Agarwal et al. 2012 [23] | RCT  nonvalvular AF |  | 54.7-69 | 70-81.5 | Hypertension: 53.1-90.8%  CAD: 33- 41%  MI: 9.6-18.0% previous stroke/TIA: 13.1-54.6%  HF: 19.7-62.3% DM: 17-39.5 systemic embolism: 4.3-5 % | 2.0-3.46 | 55-68 |
| Aguilar et al. 2005 [53] | RCT  OACs vs. placebo/control  FU >4weeks | Combination of antiplatelet +OAC  Iatrogenic cardioversion  Mitral stenosis/prosthetic cardiac valves | 74 | 69 | Hypertension: 45%  Diabetes: 15%  prior MI: 15%  prior stroke or TIA: 3-8 % | Not reported | 43-83 in the studies reporting TTR |
| Aguilar et al. 2007 [59] | RCT  Non-valvular AF | Iatrogenic cardioversion  Prosthetic valves/mitral stenosis | 45-66 | 64 to over 75 | Prior stroke or TIA: 10% | Not reported | 48-84 |
| Andersen et al. 2008 [55] | RCT  AF or flutter  FU ≥3 months | Postoperative AF  Valvular disease | Not reported | 63.3-81.5 | Not reported | Not reported | 43.7-83.5 |
| Assiri et al. 2013 [50] | RCT  Double blind  English  VKA: INR 2-3 |  | 58.7 | 71 | Not reported | 1.8-3.47 | 42-83 |
| Baker et al. 2012 [33] | RCT  Nonvalvular AF  Apixaban, dabigatran, rivaroxaban vs. warfarin or each other  outcome: stroke/SE  major bleeding |  | not reported | 69-73 | Hypertension: 70-90.8%,  CHF: 31-62.6%, DM: 21.4-40.4%, prior TIA or stroke: 17.5-54.9% | 2.1-3.48 | 55-64 |
| Briceno et al. 2015 | RCT  Nonvalvular AF  NOACs or Watchman device vs. warfarin |  | 60.3-80.6 | 70-74 | Not reported | 2.1-3.5 | 55.-70 |
| Cameron et al. 2014 [49] | RCT (phase III)  Nonvalvular AF  VKA: INR 2-3  English | Trials including patients with contraindication to anticoagulation | Not reported | 62-83 | Prior stroke 3%-55% | 1.9-3.5 | 44-83 |
| Capodanno et al. 2013 [27] | RCT (hase III)  Non-valvular AF  NOACs vs. warfarin  Both efficacy and safety outcomes reported |  | 63 | 71 | Prior stroke/ SE/TIA: 31%  Prior MI: 16%  Hypertension:  85% | 2.5  (2.1-3.5) | 58-66 |
| Chatterje et al. 2013 [42] | RCT  AF  NOACs |  | study drug: 59-83.7  control drug: 58-81.1 | 69.6-73 | Not reported | Not reported | 60-65 |
| Coleman et al. 2012 [45] | RCT  ≥18 years  AF  Report data on MGIB | Postoperative atrial fibrillation or flutter | 41-100 | 65-75 | Not reported | Not reported | Not reported |
| Cooper et al. 2006 [58] | FU >12 month |  | 32-100 | 64-80.2 | Prior stroke: 0-100% | Not reported | Not reported |
| Dogliotti et al. 2013 [28] | RCT  N >3000  intention-to-treat analysis |  | 60.3-70 | 70-73 | Previous stroke/TIA:  19.8-24 %  Hypertension:  72-90,3%  Diabetes:  23.1-40,4%  LVD or heart failure:  31.8-62.6% | Not reported | Not reported |
| Dogliotti et al. 2014 [46] | RCT (phase II or III)  VKA, ASA, clopidogrel or NOACs  Non-valvular AF  Intention-to-treat analysis | FU <1 year  Subgroup analysis  Posthoc analysis  Low-dose warfarin  Registry data | 50-100 | 64-83 | Hypertension: 31.5-90.5%  Diabetes: 4-40% prior MI: 9-19% or not reported; prior TIA or stroke: 5-55% or not reported | 2-3.47 or not reported for single studies | Not reported |
| Harenberg et al. 2012 [61] | RCT  Control: VKA adjusted (INR 2-3) |  | 60.30-64.75 | 70-73 | Not reported | ≥3: 30.22-86.95 | 55-66 |
| Hart et al. 1999 [4] | RCT  FU >3 months | Mitral stenosis/prosthetic cardiac valves | 71 | 69 | Hypertension: 45 %  prior stroke or TIA: 20% | Not reported | Not reported |
| Hart et al. 2007 [57] | RCT  Nonvalvular AF  FU ≥12 weeks | Mitral stenosis/prosthetic cardiac valves | 75 | 71 | Not reported | Not reported | Not reported |
| Holster et al. 2013 | RCT  NOAC  bleeding as outcome  indication for anticoagulation |  | 59-82 | 65-73 | Not reported | 1.8-3.5 | 45.1-66 |
| Jia et al. 2014 | RCT  NOAC vs. warfarin  AF | ximelagatran  darexaban | 57-82.9 | 70-73 | previous stroke/TIA:18-63.9%  Heart failure: 32-63%  Diabetes: 23-62.8%  Hypertension: 79-94% | 2.1-3.5 | 44-68 |
| Lega et al. 2014 | RCT  nonvalvular AF  results reported according to age, CHADS2 score, heart failure, diabetes GFR prioir exposure to VKA, prior stroke or TIA |  | Not reported | 70-73 | Not reported | Not reported | 55-64 |
| Liew et al. 2014 [29] | RCT  Patients with AF  NOAC vs. warfarin  FU >1 year  One of the following outcomes: intracranial bleeding, bleeding-,all-cause or vascular mortality |  | Approx. 66 | 70-73 | Not reported | 2.1-3.5 | 58-68.4 |
| Lin et al. 2015 | RCT (II or III)  observational studies  AF  warfarin, ASA or NOACs | warfarin at non-standard doses  triple therapy with warfarin, ASA, clopidogrel | NRSs 56%, RCTs 62% | 71.5 | Hypertension 17-94%, chronic heart failure 1-70%, diabetes mellitus 3-45%, prior TIA/ stroke 3-55% | 2.3 | Not reported |
| Lip et al. 2006 [54] | RCT  Non-valvular AF |  | Not reported | 64-80 | Not reported | Not reported | Not reported |
| Miller et al. 2012 [30] | RCT  Oral anticoagulants  AF  FU > 1 year | ximelagatran | 60.3-65 | 70-73 | Prior stroke/TIA: 19.2-62.6%  Heart failure: 31.8-62.6%  Diabetes mellitus: 23.4-40.4%  Hypertension: 78.9-90.8% | 2.1-3.5 | 55-64 |
| Providência et al. 2014 [31] | RCT (phase III)  NOACs vs. warfarin  Non-valvular AF | Phase II trials  Observational studies  Interventions (e.g. catheter ablation) as comparison | 60.3-80.6 | 70-73 | CHF: 32.0-62.5%  Hypertension: 72.2-93.5%  Diabetes:  23.3-39.9%  Prior stroke/TIA: 18.3-63.6%  Prior MI: 7.7-17.3% | 2.1-3.5 | 55-68 |
| Rong et al. 2015 | RCT  AF  NOACs vs. warfarin |  | 57-65 | 70-73 | previous stroke/TIA: 18-55%  Heart failure: 32-63%  Diabetes: 23-40%  Hypertension: 79-94% | 2.1-3.5 | 58-68 |
| Roskell et al. 2010 [47] | RCT  Patients with AF treated for the prevention of stroke  VKA, aspirin, clopidogrel, indobufen, idraparinux, triflusal, ximelagatran, and dabigatran | Phase I trials | 60-70 | 67.6-74.9 | Not reported | Not reported | 65 |
| Ruff et al. 2014 [44] | RCT (phase III)  Patients with AF  NOACs vs. warfarin |  | 60-65 | 70-73 | Previous stroke/TIA: 29% (20-55%)  Heart failure: 46% (35-63%)  Diabetes: 31% (23-40%)  Hypertension: 88% (79-94%)  Prior myocardial infarction: 15% (11-18%) | 2.1-3.5 | 65  (58-68) |
| Sardar et al. 2013 [43] | RCT  NOACs vs. conventional therapy  patients with and without prior stroke/TIA  FU >1years |  | 61-64 | 70.1-71 | Prior stroke/TIA: 100%  Diabetes: 21.4-26%  Hypertension: 77-85% | Not reported | 57.1-65 |
| Sardar et al. 2014 [26] | RCT  NOACs (vs. conventional therapy)  elderly participants |  | in AF trials 58-65 | 53,9 - 71,6 subgroup for ≥75 | Not reported | Not reported | Not reported |
| Schneeweiss et al. 2012 [51] | RCT (phase III) |  | 60.3-65 | 70-73 | Only reported for warfarin group:  prior stroke/TIA/syst embolism: 19.7-54.6%  HF: 31.9-62.3%  Diabetes: 23.4-39.5%  Hypertension: 78.9-90.8% | Only reported for warfarin group:  ≤1: 0-34%  2: 13.1-37%;  ≥3:30.2-86.9% | Only reported for warfarin group:  55-64 |
| Segal et al. 2000 [56] | RCT |  | 24-100 | 66-80 | DM: 8%-32%, CHF: 9%-71%, hypertension: 32%-58% | Not reported | Not reported |
| Senoo et al. 2015 | RCT  NOAC or warfarin  Japanese patients with AF  Follow Up >1 year |  | 76.7-82.9 | 70-73 | previous stroke/TIA: 24.8-63.8%  Heart failure: 18-41.3% | 2.0-3.27 | 60 |
| Sharma et al. 2015 [22]  (Subgroup > 75 with AF) | RCT (phase II or III)  AF and VTE  NOACs vs. VKA  FU > 3 month |  | 60.3-82.4 | Only data for patients >75 included | Not reported | 1.8-3.5 | 55-65 |
| Taylor et al. 2001 [60] | Non-rheumatic AF  Antiplatelet or anticoagulation >1 year | Combined use of anticoagulation/antiplatelet | Not reported | 64-80 | Prior TIA/stroke: 5.5-50% | Not reported | Not reported |
| Testa et al. 2012 [32] | RCT  Comparison to a Vit. K antagonist  FU >1year  Intention-to-treat analysis |  | 35.5-64.3 | 70-73 | Prior stroke/TIA/SE: 19.2-54.9%  Previous MI: 14.5-16.9%  Diabetes 23.1-40.4% | Not reported | 55-65 |
| Verdecchia et al. 2015 [48] | RCT (phase III)  NOACs vs. warfarin  Non-valvular AF  Intention-to-treat analysis  FU >1 year |  | 60-65 | 70-73 | Prior stroke/TIA: 20.0-54.8  HF: 32.0-62.5  Diabetes: 23.3-40.0  Hypertension: 78-9-90.5% | 2.1-3.5 | 55-65 |

Note: AF=atrial fibrillation, ASA=aspirin, CAD=coronary artery disease, CHF=congestive heart failure, DM=diabetes mellitus, FU= Follow up, GFR= glomerular filtration rate, HF=heart failure, LVD=left ventricular diastolic, MGIB=major gastrointestinal bleeding, MI=myocardial infarction, NOAC=new oral anticoagulants, OAC=oral anticoagulation, RCT=randomised controlled trial, SE=systemic embolism, TIA= transient ischemic attack, VKA= vitamin K antagonists, VTE= venous thromboembolism
