# Supplementary material for: Effectiveness and safety of vitamin K antagonists and new anticoagulants in the prevention of thromboembolism in atrial fibrillation in older adults – a systematic review of reviews and the development of recommendations to reduce inappropriate prescribing
Source: BMC Geriatr. 2017 Oct 16;17(Suppl 1):223. doi: 10.1186/s12877-017-0573-6 (PMC5647558; doi:10.1186/s12877-017-0573-6)
Supplement: Supplementary file 3 — Dates and databases of Systematic Review searches. (DOCX 91 kb) [file 12877_2017_573_MOESM3_ESM.docx]

Additional file 3: Table S2 - Dates and databases of Systematic Review searches

| **Source** | **Dates** | **Databases** |
| --- | --- | --- |
| Adam et al. 2012 [[27](#_ENREF_27)] | January 2001 to July 2012 | MEDLINE, EMBASE, Cochrane,  FDA, database for adverse event reports |
| Agarwal et al. 2012 [[23](#_ENREF_23)] | N.A. | MEDLINE, EMBASE, Cochrane |
| Aguilar et al. 2005 [[30](#_ENREF_30)] | June 2004  *The Cochrane Library* Issue 4, 2004  1966 to June 2004 | Cochrane Stroke Group Trials Register,  CENTRAL,  MEDLINE,  Manual search |
| Aguilar et al. 2007 [[40](#_ENREF_40)] | June 2006  *The Cochrane Library* Issue 2, 2006  1966 to June 2006  1980 to June 2006 | Cochrane Stroke Group Trials Register,  CENTRAL,  MEDLINE,  EMBASE,  Manual search |
| Andersen et al. 2008 [[33](#_ENREF_33)] | 1966 to November 2007 | MEDLINE, EMBASE, Cochrane Library, SveMed |
| Assiri et al. 2013 [[38](#_ENREF_38)] | January 1, 1991 to August 31, 2012 | MEDLINE, EMBASE, Cochrane Collaboration |
| Baker et al. 2012 [[49](#_ENREF_49)] | January 1950 to February 2012 | MEDLINE, CENTRAL, ClinicalTrials.gov Website, manual search |
| Briceno 2015 [[25](#_ENREF_25)] | January 1990 to April 2015 | PubMed, CENTRAL, EMBASE, ClinicalTrials.gov Website, Google Scholar databases |
| Cameron et al. 2014 [[42](#_ENREF_42)] | 1988 to January 23, 2014 | MEDLINE, MEDLINE In-Process, other Non-Indexed Citations, PubMed, EMBASE, BIOSIS, CENTRAL |
| Capodanno et al. 2013 [[43](#_ENREF_43)] | January 1980 to August 2011 | MEDLINE, Cochrane |
| Chatterje et al. 2013 [[59](#_ENREF_59)] | Inception to December 1, 2012 | ClinicalTrials.gov Website, CINAHL, CENTRAL, EBSCO |
| Coleman et al. 2012 [[31](#_ENREF_31)] | January 1950 through November 2010  Inception to November 2010 | MEDLINE,  CENTRAL,  manual search |
| Cooper et al. 2006 [[37](#_ENREF_37)] | January 2000 and March 2005 | CENTRAL, MEDLINE |
| Dogliotti et al. 2013 [[44](#_ENREF_44)] | August 2000 to October 2012 | MEDLINE, EMBASE, Cochrane database of Systematic Reviews |
| Dogliotti et al. 2014 [[36](#_ENREF_36)] | Inception to May 2013 | MEDLINE, EMBASE, Cochrane database of Systematic Reviews |
| Harenberg et al. 2012 [[67](#_ENREF_67)] | N.A. | MEDLINE, EMBASE, ClinicalTrials.gov Website, manual search |
| Hart et al. 1999 [[4](#_ENREF_4)] | 1990 to 1997 | MEDLINE |
| Hart et al. 2007 [[35](#_ENREF_35)] | 1966 to March 2007 | OVID and MEDLINE, Cochrane Stroke Group Trials Register |
| Holster 2013 [[29](#_ENREF_29)] | Inception to July 2012 | MEDLINE, EMBASE, CENTRAL |
| Jia 2014 [[50](#_ENREF_50)] | Inception to December 2013* | EMBASE, MEDLINE, Science Citation Index, Cochrane Library,  Expanded, ProQuest, manual search |
| Lega 2014 [[26](#_ENREF_26)] | Inception to October 2012 | MEDLINE, EMBASE |
| Liew et al. 2014 [[45](#_ENREF_45)] | January 1, 2009 to November 30, 2013 | PubMed, ClinicalTrials.gov Website |
| Lin 2015 [[39](#_ENREF_39)] | Inception to May 1, 2015 | PubMed, EMBASE, Cochrane Library |
| Lip et al. 2006 [[32](#_ENREF_32)] | N.A. | CENTRAL, BIOSIS, EMBASE, MEDLINE |
| Miller et al. 2012 [[46](#_ENREF_46)] | Inception to July 2011 | Cochrane Library, EMBASE, MEDLINE, Science Citation Index Expanded, ProQuest |
| Providência et al. 2014 [[47](#_ENREF_47)] | Inception to November 21, 2013 | MEDLINE, EMBASE, CENTRAL |
| Rong 2015 [[62](#_ENREF_62)] | Inception to July 2014* | Cochrane Library, EMBASE, MEDLINE, Science Citation Index Expanded, ProQuest, ClinicalTrials.gov Website, manual search |
| Roskell et al. 2010 [[63](#_ENREF_63)] | Inception to August 2009 | Cochrane database of Systematic  Reviews, CENTRAL, database of Abstracts of Reviews of Effectiveness, MEDLINE, MEDLINE In-Process, EMBASE, BIOSIS |
| Ruff et al. 2014 [[82](#_ENREF_82)] | January 1, 2009 to November 19, 2013 | MEDLINE |
| Sardar et al. 2013 [[60](#_ENREF_60)] | January 2001 to September 2012 | PubMed, CENTRAL, EMBASE, CINAHL |
| Sardar et al. 2014 [[28](#_ENREF_28)] | January 1, 2001 to March 30, 2013 | PubMed, Cochrane Library, EMBASE, Web of Science, CINAHL |
| Schneeweiss et al. 2012 [[65](#_ENREF_65)] | Inception to October 2011* | PubMed, ClinicalTrials.gov Website, FDA |
| Segal et al. 2000 [[34](#_ENREF_34)] | 1948 to May 1998 | CENTRAL, MEDLINE |
| Senoo 2015 [[61](#_ENREF_61)] | 2004 to July 2014 | PubMed, CINAHL, SCOPUS, Cochrane Library |
| Sharma et al. 2015 [[22](#_ENREF_22)] | November 22, 1993 to November 22, 2013 | MEDLINE, EMBASE, CENTRAL |
| Taylor et al. 2001 [[41](#_ENREF_41)] | 1966 to December 1999 | Cochrane Library, MEDLINE, EMBASE, CINAHL, Sigle |
| Testa et al. 2012 [[48](#_ENREF_48)] | Appendix A for further detail  2008–2011 | PubMed, Google Scholar, Cochrane Library, SCOPUS,  manual search |
| Verdecchia et al. 2015 [[64](#_ENREF_64)] | through February 2014 | MEDLINE, ISI Web of Knowledge, SCOPUS |

Notes: *=supposed (not exactly mentioned in the text); CENTRAL = Cochrane Central Register of Controlled Trials; FDA = U.S. Food and Drug Administration; the term *manual search* included inter alia Atrial Fibrillation Collaboration, contacting experts working in the field to identify unpublished and ongoing trials, the scientific session abstracts in Circulation, Journal of the American College of Cardiology, European Heart Journal, American Journal of Cardiology, conference proceedings of the American College of Cardiology, American Heart Association, European Society of Cardiology scientific sessions
