# Supplementary material for: Effectiveness and safety of vitamin K antagonists and new anticoagulants in the prevention of thromboembolism in atrial fibrillation in older adults – a systematic review of reviews and the development of recommendations to reduce inappropriate prescribing
Source: BMC Geriatr. 2017 Oct 16;17(Suppl 1):223. doi: 10.1186/s12877-017-0573-6 (PMC5647558; doi:10.1186/s12877-017-0573-6)
Supplement: Supplementary file 4 — Summary of study findings for the comparison of VKA vs. placebo. Table S4. Summary of study findings for the comparison of VKA vs. antiplatelets. (DOCX 28 kb) [file 12877_2017_573_MOESM4_ESM.docx]

Additional file 4

Table S3 - Summary of study findings for the comparison of VKA vs. placebo

|  | **N studies** | **Effect- model** | **Effect measure** | **Stroke/SE** | **Ischemic**  **stroke** | **mortality** | **major bleeding** | **intracranial bleeding** | **gastrointestinal bleeding** | **myocardial**  **infarction** |
| --- | --- | --- | --- | --- | --- | --- | --- | --- | --- | --- |
| Aguilar et al. 2005 [53] | 5 | FEM | OR | strokes  0.39  (0.26-0.59) | 0.34  (0.23-0.52) | 0.69  (0.50-0.94) | EC 1.07  (0.53-2.12) | 2.38  (0.54-10.5) | - | 0.87  (0.32-2.42) |
| Andersen et al. 2008 [55] | 4 | FEM | OR | Only SE:  0.29 (0.08-1.07) | - | - | 3.01 (1.31-6.92) | - | - | - |
| Coleman et al. 2012 [45] | 4 | Not stated | OR | - | - | - | - | - | 3.21  (1.32-7.82) | - |
| Hart et al. 1999 [4] | 6 | Not stated | RRR/RR | RRR  62% (48 to 72%) | RRR  65% (52 to 74%) | RRR  26% (4 to 43%) | only EC  RR 2.4 (1.2-4.6) | No significant difference | - | - |
| Hart et al. 2007 [57] | 6 | REM | RRR | 64% (49 to 74%) | 67% (54 to 77%) | 26% (3 to 43%) | EC -66 (-235 to 18%) | - | - | - |
| Lip et al. 2006 [54] | 6 | FEM | RR | - | 0.33  (0.24-0.45) | 0.69  (0.53-0.89) | 2.22 (1.22-4) | - | - | - |
| Segal et al. 2000 [56] | 6 | REM | OR | 0.30 (0.19-0.48) | - | 0.62 (0.38-1.02) | 1.90 (0.89-4.04) | - | - | - |
| Assiri et al. 2013 [50] | 4 /21 | Not stated | RR | 0.33 (0.24-0.48) | - | 0.67 (0.50-0.89) | 3.12 (1.05-9.96) | - | - | - |
| Cooper et al. 2006 [58] | 6 | REM | RR | - | 0.35  (0.24-0.52)  0.35  (0.19-0.60) | - | No significant difference | - | - | - |
| Dogliotti et al. 2014 [46] | 20 | Not stated | OR | 0.39 (0.30-0.53 | 0.34 (0.24-0.48) | 0.60 (0.43-0.77) | OR 3.63 (1.84-9.06) | - | - | - |
| Lin et al. 2015 | 49 | Not stated | RR | no treatment  0.66 (0.49-0.91)* | no treatment  0.65 (0.43-0.97)* | no treatment  0.62 (0.44-0.88)* | no treatment  1.14 (0.46-2.78)* | no treatment  1.25 (0.98-1.59)* | no treatment  5.88 (0.29-100)* | no treatment  0.59 (0.19-1.89)* |

Note: EC=extracranial, FEM=fixed effects model, OR=Odds ratio, PL=placebo, REM=random effects model, RR=risk ratio, RRR= relative risk reduction, WAR=warfarin

*reciprocal was calculated by the review team

Table S4 - Summary of study findings for the comparison of VKA vs. antiplatelets

|  | **N studies** | **Effect- model** | **Effect measure** | **Stroke/SE** | **Ischemic**  **stroke** | **mortality** | **major bleeding** | **intracranial bleeding** | **gastrointestinal bleeding** | **myocardial**  **infarction** |
| --- | --- | --- | --- | --- | --- | --- | --- | --- | --- | --- |
| Aguilar et al. 2007 [59] | 8 | FEM | OR | 0.68  (0.54-0.85) | 0.53  (0.41-0.68) | 0.99  (0.83-1.18) | EC 0.97  (0.74-1.28) | 1.98  (1.20-3.28) | - | 0.69  (0.47-1.01) |
| Andersen et al. 2008 [55] | 9 | FEM | OR | Only SE:  0.50 (0.33 - 0.75) | - | - | 1.07 (0.85 - 1.34) | - | - | - |
| Coleman et al. 2012 [45] | 7 | Not stated | OR | - | - | - | - | - | 1.92  (1.08-3.41) | - |
| Hart et al. 1999 [4] | 5 |  | RRR/RR | 36% (14 to 52%) | 46% (27 to 60%) | 8% (-21 to 30%) | EC RR2.0 (1.2-3.4) | RR 2.1 (1.0-4.6) | - | - |
| Hart et al. 2007 [57] | 9 | REM | RRR | 37% (23 to 48%) | 52% (41to 62%) | 9% (-19 to 30) | EC -70% (-234 to 14) | -128% (-399 to -4) | - | - |
| Lip et al. 2006 [54] | 5 | FEM | RR | - | 0.59  (0.40-0.86) | 0.87  (0.67-1.13) | 1.72 (1.03-2.86)0.58  (0.35-0.97) | - | - | - |
| Segal et al. 2000 [56] | 3 | REM | OR | 0.64 (0.43-0.96) | - | 0.96 (0.58-1.58) | 1.60 (0.77-3.35) | - | - | - |
| Taylor et al. 2001 [60] | 6 | FEM | OR | Fatal 0.74  (0.39 to 1.40)  Non-fatal  0.68 (0.46-0.99) | - | 0.94 (0.72-1.21) | 1.45  (0.93 to 2.27) | - | - | 0.83 (0.46-1.50) |
| Assiri et al. 2013 [50] | ? | Not stated | RR | ASA:  0.43 (0.33-0.57)  ASA+clopidogrel: 0.60 (0.42-0.85) | - | ASA:  0.85 (0.70-1.02)  ASA+clopidogrel: 0.90 (0.70-1.18) | ASA:  1.42 (0.61-2.69)  ASA+clopidogrel: 0.95 (0.27-2.78) | ASA:  1.95 (0.45-9.29)  ASA+clopidogrel: 1.03 (0.15-7.59) | - | - |
| Cameron et al. 2014 [49] | 12/15 | FEM | OR | ASA (≤100mg)  0.53 (0.36-0.79)  ASA (100-300mg)  0.74 (0.40-1.35)  low dose ASA+clopidogrel  0.52 (0.38-0.70) | - | - | ASA (≤100mg)  0.95 (0.53-1.67)  ASA (100-300mg)  0.56 (0.18-1.61)  low dose ASA+clopidogrel  0.91 (0.68-1.20) | - | - | - |
| Cooper et al. 2006 [58] | 19 |  | RR | - | RR 1.85 (1.25-2.58) | - | Not significant | - | - | - |
| Dogliotti et al. 2014 [46] | 9 | FEM | OR | 0.51  (0.41- 0.65) | 0.43  (0.33- 0.58) | 0.77  (0.58- 0.92) | 1.71  (1.05- 3.23) | - | - | - |
| Lin et al. 2015 | 49 | Not stated | RR | no treatment  0.78 (0.54-1.14)* | no treatment  0.77 (0.44-1.33)* | no treatment  0.82 (0.56-1.22)* | no treatment  0.85 (0.38-1.89)* | no treatment  0.91 (0.23-3.70)* | no treatment  0.45 (0.08-2.50)* | no treatment  0.86 (0.43-1.75)* |

Note: EC=extracranial, FEM=fixed effects model, OR=Odds ratio, REM=random effects model, RR=risk ratio, RRR= relative risk reduction
