# Supplementary material for: Effectiveness and safety of vitamin K antagonists and new anticoagulants in the prevention of thromboembolism in atrial fibrillation in older adults – a systematic review of reviews and the development of recommendations to reduce inappropriate prescribing
Source: BMC Geriatr. 2017 Oct 16;17(Suppl 1):223. doi: 10.1186/s12877-017-0573-6 (PMC5647558; doi:10.1186/s12877-017-0573-6)
Supplement: Supplementary file 5 — Heterogenity. (DOCX 52 kb) [file 12877_2017_573_MOESM5_ESM.docx]

Additional file 5: Table S5 - Heterogenity

| Author | N studies | Stroke/SE | Ischemic  stroke | haemorrhagic  stroke | mortality | major bleeding | intracranial bleeding | gastrointestinal bleeding | myocardial  infarction |
| --- | --- | --- | --- | --- | --- | --- | --- | --- | --- |
| Adam et al. 2012 [[25](#_ENREF_25)] | 3 | --- | not reported | not reported | not reported | --- | --- | --- | --- |
| Baker et al.2012 [[45](#_ENREF_45)] | 4 | 28.5% | 0% | 52.1% | 0% | 80.6% | --- | 82.5% | --- |
| Briceno et al. [[76](#_ENREF_76)] | 5 | 65% | --- | --- | 0% | 88% | --- | --- | --- |
| Capodanno et al. 2013 [[39](#_ENREF_39)] | 3 | 0% | 0% | 59% | 0% | 86% | 70% | 91% | 76% |
| Dogliotti et al. 2013 [[40](#_ENREF_40)] | 5 | 62% | not reported | not reported | 0% | not reported | --- | --- | --- |
| Holster et al. 2013 [[77](#_ENREF_77)] | 8 | --- | --- | --- | --- | 82% | --- | 61% | --- |
| Jia et al. [[78](#_ENREF_78)] | 5 | 73% | not reported | not reported | not reported | 90.8% | not reported | not reported | not reported |
| Lega et al. [[79](#_ENREF_79)] | 3 | >75years: 0%  <75years: 25.1% | --- | --- | --- | >75years: 0.87%  <75years: 33.1% | --- | --- | --- |
| Liew et al. 2014 [[41](#_ENREF_41)] | 4 | --- | --- | --- | 0% | --- | 53% | --- | --- |
| Miller et al. 2012 [[42](#_ENREF_42)] | 3 | 55.9% | 0% | 52.2% | not reported | 87.2% | 54.9% | 82.5% | not reported |
| Providência et al. 2014 [[43](#_ENREF_43)] | 7 | 46% | 58% | Not stated | 0% | 82% | 58% | 73% | 56% |
| Rong et al. 2015 [[81](#_ENREF_81)] | 4 | --- | --- | --- | --- | 92.3%  high dose: 82.5%  low dose: 95.9% | 57.6%  high dose: 33.8%  low dose: 0% | 85.1%  high dose: 72.8%  low dose: 88.2% | --- |
| Ruff et al. 2014 [[24](#_ENREF_24)] | 4 | 47% | 32% | 34% | 0% | 83% | 32% | 74% | 48% |
| Sardar et al. 2013 [[55](#_ENREF_55)] | 3 | 0% | --- | 62% | 18% | 52% | 64% | 53% | --- |
| Senoo et al. [[82](#_ENREF_82)] | 3 | 0% | --- | --- | --- | 64% | 0% | 0% | --- |
| Testa et al. 2012 [[44](#_ENREF_44)] | 3 | 22% | 37% | 48% | 0% | EC 73% | --- | --- | 73% |

Note: EC: extracranial
