# Supplementary material for: Effectiveness and safety of vitamin K antagonists and new anticoagulants in the prevention of thromboembolism in atrial fibrillation in older adults – a systematic review of reviews and the development of recommendations to reduce inappropriate prescribing
Source: BMC Geriatr. 2017 Oct 16;17(Suppl 1):223. doi: 10.1186/s12877-017-0573-6 (PMC5647558; doi:10.1186/s12877-017-0573-6)
Supplement: Supplementary file 6 — Eventrates NOACs and VKA. (DOCX 24 kb) [file 12877_2017_573_MOESM6_ESM.docx]

| **Additional file 6: Table S6: Eventrates NOACs and VKA** | |  |  |
| --- | --- | --- | --- |
| ***Author*** | ***Eventrate NOACs*** | ***Eventrate VKA*** | ***Reported effect measure*** |
| **Stroke/SEE** | | | |
| Baker et al.2012 | 615/22377 | 770/22263 | RR 0.797 (99% CI 0.695-0.914) |
| Briceno et al. 2015 | 1487/43808  subgroup >75 years:  544/16280 | 1129/29866  subgroup >75 years:  594/13804 | OR 0.84 (0.72-0.97)  subgroup >75 years:  OR 0.77 (0.68-0.87) |
| Capodanno et al. 2013 | 797/28292 | 770/22193 | OR 0.82 (0.74-0.91) |
| Dogliotti et al. 2013 | Not reported | Not reported | RR 0.82 (0.69-0.98) |
| Jia et al. | 1487/43000  high dose: 922/29951  low dose: 565/13049 | 1665/42926  high dose: 1129/29868  low dose: 536/13058 | RR 0.86 (0.75-0.99)  high dose: RR 0.80 (0.71-0.91)  low dose: RR 1.03 (0.84-1.27) |
| Lega et al. | Not reported | Not reported | >75years: RR 0.77 (0.67-0.89)  <75years: RR 0.83 (0.71-0.96) |
| Miller et al. 2012 | 615/22277 | 773/22193 | RR 0.78 (0.67-0.92) |
| Providência et al. 2014 | 1250/46662 | 1046/33531 | RR 0.84 (0.74-0.95) |
| Ruff et al. 2014 | 911/29312 | 1107/29229 | RR 0.81 (0.73-0.91) |
| Sardar et al. 2013 | 358/7876 | 415/7846 | OR 0.85 (0.74-0.99) |
| Senoo et al. | 14/1016 | 28/920 | RR 0.45 (0.24-0.85) |
| Testa et al. 2012 | 697/28292 | 757/28215 | OR 0.92 (0.83-1.02) |
| **Ischemic stroke** | | | |
| Adam et al. 2012 | 409/22257 | 458/22185 | RR 0.89 (0.78-1.02) |
| Baker et al.2012 | 422/22257 | 478/22185 | RR 0.880 (99%CI 0.742-1.044) |
| Capodanno et al. 2013 | 588/28292 | 489/22193 | OR 0.93 (0.82-1.05) |
| Dogliotti et al. 2013 | Not reported | Not reported | RR 0.87 (0.72-1.06) |
| Jia et al. | high dose: 714/29951  low dose: 492/13049 | high dose: 765/29868  low dose: 377/13058 | high dose 0.93 (0.84-1.03)  low dose 1.13 (1.14-1.49) |
| Miller et al. 2012 | 429/22257 | 489/22185 | RR 0.87 (0.77-0.99) |
| Providência et al. 2014 | 1203/46644 | 784/33525 | RR 0.97 (0.83-1.14) |
| Ruff et al. 2014 | 665/29292 | 724/29221 | RR 0.81 (0.73-0.91) |
| Testa et al. 2012 | 581/28292 | 620/28215 | OR 0.92 (0.83-1.02) |
| **Haemorrhagic stroke** | | | |
| Adam et al. 2012 | 81/22257 | 173/22185 | RR 0.48 (0.36-0.62) |
| Baker et al.2012 | 81/22257 | 179/22185 | RR 0.445 (99%CI 0.269-0.768) |
| Capodanno et al. 2013 | 95/28292 | 173/22193 | OR 0.44 (0.30-0.66) |
| Dogliotti et al. 2013 | Not reported | Not reported | RR 0.49 (0.35-0.70) |
| Jia et al. | high dose: 137/29951  low dose: 44/13049 | high dose: 271/29868  low dose: 135/13058 | high dose: RR 0.50 (0.41-0.62)  low dose: RR 0.33 (0.23-0.46) |
| Miller et al. 2012 | 81/22257 | 173/22185 | RR 0.45 (0.31-0.68) |
| Ruff et al. 2014 | 130/29292 | 263/29221 | RR 0.49 (0.38-0.64) |
| Sardar et al. 2013 | 41/7876 | 97/7846 | OR 0.37 (0.19-0.72) |
| Testa et al. 2012 | 95/28292 | 218/28215 | OR 0.43 (0.34-0.55) |
| **Mortality** | | | |
| Adam et al. 2012 | 1249/22257 | 1406/22185 | RR 0.88 (0.82-0.96) |
| Baker et al.2012 | 1249/22307 | 1406/22228 | RR 0.874 (99%CI 0.803-0.974) |
| Briceno et al. 2015 | 3212/42978 | 2250/29858 | OR 0.89 (0.84-0.94) |
| Capodanno et al. 2013 | 1695/28292 | 1406/22193 | OR 0.88 (0.82-0.95) |
| Dogliotti et al. 2013 | Not reported | Not reported | RR 0.91 (0.85-0.96) |
| Jia et al. | high dose: 2238/29951  low dose: 1183/13049 | high dose: 2479/29868  low dose: 1326/13058 | high dose: RR 0.90 (0.85-0.95)  low dose: RR 0.89 (0.83-0.96) |
| Liew et al. 2014 | 3205/42341 | 2245/29221 | RR 0.89 (0.85–0.94 |
| Miller et al. 2012 | Not reported | Not reported | RR 0.88 (0.82-0.95) |
| Providência et al. 2014 | 3406/46642 | 2452/33523 | RR 0.90 (0.86-0.95) |
| Ruff et al. 2014 | 2022/29292 | 2245/29221 | RR 0.90 (0.85–0.95) |
| Sardar et al. 2013 | 602/7876 | 658/7876 | OR 0.90 (0.79-1.02) |
| Testa et al. 2012 | 1632/28292 | 1796/28215 | OR 0.90 (0.84-0.96) |
| **Major bleeding** | | | |
| Baker et al. 2012 | 1097/22375 | 1245/22269 | RR 0.878 (99%CI 0.664-1.160) |
| Briceno et al. | 2161/42943 | 1823/29850 | OR 0.794 (0.647-0.973) |
| Capodanno et al. 2013 | 1419/28292 | 1245/22193 | OR 0.85 (0.69-1.05) |
| Dogliotti et al. 2013 | Not reported | Not reported | RR 0.84 (0.70-1.00) |
| Holster et al. 2013 | Not reported | Not reported | OR 0.93 (0.75-1.16) |
| Jia et al. | 2117/42943  high dose: 1541/29926  low dose: 576/13017 | 2702/42884  high dose: 1799/29850  low dose: 921/13034 | RR 0.78 (0.64-0.94)  high dose: RR 0.86 (0.74-0.99)  low dose: RR 0.63 (0.38-1.04) |
| Lega et al. | Not reported | Not reported | >75years: RR 0.90 (0.82-1.00)  <75years: RR 0.73(0.65-0.81) |
| Miller et al. 2012 | 1121/22275 | 1269/22199 | RR 0.88 (0.71-1.09) |
| Providência et al. 2014 | 2253/46606 | 1948/33516 | RR 0.79 (0.67-0.93) |
| Rong et al. 2015 | 2091/42304  high dose: 1515/29287  low dose: 576/13017 | 2690/42245  high dose: 1769/29211  low dose: 321/13034 | RR 0.77 (0.63-0.95)  high dose: RR 0.86 (0.73-1.01)  low dose: RR 0.63 (0.38-1.04) |
| Ruff et al. 2014 | 1541/29292 | 1802/29221 | RR 0.86 (0.73-1.00) |
| Sardar et al. 2013 | 422/7876 | 483/7846 | RR 0.84 (0.69-1.03) |
| Senoo et al. | 43/1017 | 56/922 | RR 0.66 (0.29-1.47) |
| Testa et al. 2012 | 1256/28292 | 1272/28215 | EC OR 0.98 (0.91-1.07) |
| **Intracranial bleeding** | | | |
| Capodanno et al. 2013 | 170/28290 | 293/22199 | OR 0.46 (0.38-0.55) |
| Jia et al. | high dose: 209/29926  low dose: 68/13017 | high dose: 435/29850  low dose: 219/13034 | high dose: RR 0.48 (0.41-0.56)  low dose: RR 0.31 (0.24-0.41) |
| Liew et al. 2014 | 272/42304 | 425/29211 | RR 0.42 (0.34-0.53) |
| Miller et al. 2012 | 143/22275 | 293/22199 | RR 0.49 (0.36-0.66) |
| Providência et al. 2014 | 295/46607 | 455/33515 | RR 0.49 (0.37-0.63) |
| Rong et al. 2015 | 272/42304  high dose: 204/29287  low dose: 68/13017 | 644/42245  high dose: 425/29211  low dose: 219/13034 | RR 0.42 (0.34-0.52)  high dose: RR 0.48 (0.39-0.59)  low dose: RR 0.31 (0.24-0.41) |
| Ruff et al. 2014 | 204/29287 | 425/29221 | RR 0.48 (0.39–0.59) |
| Sardar et al. 2013 | 68/7876 | 147/7846 | RR 0.42 (0.25-0.70) |
| Senoo et al. | 7/1017 | 17/922 | RR 0.46 (0.18-1.16) |
| **Gastrointestinal bleeding** | | | |
| Baker et al.2012 | 511/22275 | 393/22199 | RR 1.254 (99%CI 0.827-1.901) |
| Capodanno et al. 2013 | 644/28290 | 291/22199 | OR 1.68 (1.03-2.72) |
| Holster et al. 2013 | Not reported | Not reported | OR 1.21 (0.91-1.61) |
| Jia et al. | high dose: 635/29926  low dose: 262/13017 | high dose: 511/29850  low dose: 310/13034 | high dose: RR 1.24 (1.10-1.39)  low dose: RR 0.52 (0.19-1.00) |
| Miller et al. 2012 | 511/22275 | 393/22199 | RR 1.25 (0.91-1.72) |
| Providência et al. 2014 | 1028/44646 | 613/31554 | RR 1.07 (0.86-1.34) |
| Rong et al. 2015 | 890/42304  high dose: 628/29287  low dose: 262/13017 | 812/42245  high dose: 502/29211  low dose: 310/13034 | RR 1.10 (0.86-1.41)  high dose: RR 1.25 (0.99-1.57)  low dose: RR 0.87 (0.54-1.40) |
| Ruff et al. 2014 | 751/29287 | 591/29221 | RR 1.25 (1.01–1.55) |
| Sardar et al. 2013 | 108/4122 | 88/4132 | RR 1.17 (0.76-1.80) |
| Senoo et al. | 13/1017 | 22/922 | RR 0.52 (0.25-1.08) |
| **Myocardial infarction** | | | |
| Capodanno et al. 2013 | 366/28292 | 291/22193 | OR 0.99 (0.71–1.38) |
| Jia et al. | high dose: 434/29951  low dose: 255/13049 | high dose: 444/29868  low dose: 204/13058 | high dose 0.97 (0.85-1.11)  low dose 1.25 (1.04-1.50) |
| Miller et al. 2012 | 288/22257 | 303/22185 | RR 0.96 (0.73-1.26) |
| Providência et al. 2014 | 741/46641 | 495/33524 | RR 1.01 (0.83-1.24) |
| Ruff et al. 2014 | 413/29292 | 432/29221 | RR 0.97 (0.78–1.20) |
| Testa et al. 2012 | 366/28292 | 354/28215 | OR 1.03 (0.89-1.20) |
|  |  |  |  |
