# Supplementary material for: Effectiveness and safety of vitamin K antagonists and new anticoagulants in the prevention of thromboembolism in atrial fibrillation in older adults – a systematic review of reviews and the development of recommendations to reduce inappropriate prescribing
Source: BMC Geriatr. 2017 Oct 16;17(Suppl 1):223. doi: 10.1186/s12877-017-0573-6 (PMC5647558; doi:10.1186/s12877-017-0573-6)
Supplement: Supplementary file 7 — Recommendation. (DOCX 16 kb) [file 12877_2017_573_MOESM7_ESM.docx]

| **Recommendations** | **Strength of the recommendation** | **Quality of the evidence** | **Evidence base** |
| --- | --- | --- | --- |
| Consider to switch from vitamin K antagonists to a novel oral anticoagulants (apixaban, rivaroxaban, edoxaban, dabigatran) in patients with atrial fibrillation and a lower time in therapeutic range of the target INR than 55 %. Novel oral anticoagulants appear to decrease the rate of hemorrhagic stroke and intracranial bleeding compared to warfarin and may be associated with a lower mortality, but also may increase the rate of gastrointestinal bleeding. The rate of ischemic stroke appears to be equal for both treatments.  Patients with severe renal impairment should be excluded from this recommendation and not switch to a NOAC. | Weak  Reason: Although the outcomes were considered to be critical for decision making, the quality of the evidence was moderate which led to a weak strength of the recommendation | Moderate  It was considered to downgrade the quality of the evidence because of indirectness of the results as there is no trial that evaluated the effect of switching from vitamin K antagonists to a novel oral anticoagulant | Ten SRs (Adam et al. 2012, Baker et al.2012, Capodanno et al. 2013, Dogliotti et al. 2013, Liew et al. 2014, Miller et al. 2012, Providência et al. 2014, Ruff et al. 2014, Sardar et al. 2013, Testa et al. 2012)  of interest:  3 guidelines (NICE, ESC,AHA/ACC/HRS) |

Additional file 7: Table S7 - Recommendation
